# Convergence and Segregation of Excitatory and Inhibitory Afferents in the Paraventricular Thalamic Nucleus

**DOI:** 10.1523/JNEUROSCI.0539-25.2025

**Published:** 2025-09-09

**Authors:** László Biró, Zsolt Buday, Kata Kóta, Szabolcs Lőrincz, László Acsády

**Affiliations:** ^1^Lendület Laboratory of Thalamus Research, HUN-REN Institute of Experimental Medicine, Budapest 1083, Hungary; ^2^János Szentágothai Doctoral School of Neurosciences, Semmelweis University, Budapest 1083, Hungary

**Keywords:** axonal density, calretinin, GABAergic, glutamatergic, paraventricular thalamus, viral tracing

## Abstract

The paraventricular thalamic nucleus (PVT) integrates subcortical signals related to arousal, stress, addiction, and anxiety with top-down cortical influences. Increases or decreases in PVT activity exert profound, long-lasting effects on behavior related to motivation, addiction, and homeostasis. Yet the sources of its subcortical excitatory and inhibitory afferents, their distribution within the PVT, and their integration with layer-specific cortical inputs remain unclear. Using transgenic male and female mice selective for GABAergic and glutamatergic neurons, or for different cortical layers, we found that the input organization of PVT is unique among thalamic nuclei. PVT received subcortical GABAergic and glutamatergic inputs from multiple, distinct hypothalamic and brainstem regions. Most regions provided either excitatory or inhibitory afferents; however, subcortical inputs with dual components have also been found. Most of these subcortical inputs selectively targeted the core region of the PVT that contained large number of densely packed calretinin-positive (CR+) neurons. Cortical afferents to PVT displayed layer-specific segregation. Layer 5 neurons of the medial prefrontal cortex preferentially innervated the CR+ core, whereas layer 6 input was more abundant in the transition zone between PVT and the mediodorsal nucleus. These findings demonstrate extensive convergence of excitatory and inhibitory inputs from diverse subcortical sources, selectively, in a sharply delineated CR+ core region of PVT which is also under strong top-down control from layer 5. This unique organization may explain why the CR+ PVT core serves as a critical bottleneck in the subcortex–cortex communication involved in affective behavior.

## Significance Statement

The paraventricular thalamic nucleus (PVT) is a critical hub that integrates diverse neural signals controlling arousal, emotion, and motivation. This integration depends on which brain regions excite or inhibit PVT and whether these inputs converge or segregate. The present study reveals that the PVT receives distinct excitatory and inhibitory inputs from multiple subcortical and cortical regions. Most afferents converge in a sharply delineated core region of PVT. The work offers new insights into how structural organization of thalamus can explain its influence on behavior like stress adaptation, craving, or affective behavior. These findings can lead to novel understanding of PVT's function and guide future research to study therapeutic strategies aimed at restoring balanced neural dynamics in neuropsychiatric disorders.

## Introduction

The paraventricular nucleus of the thalamus (PVT) represents a critical bottleneck in the brainstem–cortex communication involving affective behaviors. Initially regarded as a relay for the reticular activating system (Steriade and Glenn, 1982), the PVT is now acknowledged as a complex regulator of emotional, motivational, or cognitive processes (Van der Werf et al., 2002; Hsu et al., 2014; Kirouac, 2015; McGinty and Otis, 2020; McNally, 2021; Penzo and Gao, 2021). Understanding the balance between excitatory and inhibitory inputs in the PVT is crucial for unravelling its role in behavior regulation and identifying therapeutic targets (Kolaj et al., 2014; Otis et al., 2019; Jász et al., 2025).

Classical anatomical tracing studies demonstrated extensive projections to the PVT from distinct subcortical sources (Cornwall and Phillipson, 1988a; Chen and Su, 1990; Krout and Loewy, 2000a,b; Hsu and Price, 2009; Li and Kirouac, 2012). These afferents play a crucial role in modulating a wide range of behaviors, including anxiety, stress responses, feeding, addiction, arousal, and reward processing (Betley et al., 2013; Livneh et al., 2017; Millan et al., 2017; Beas et al., 2018; Ren et al., 2018; Otis et al., 2019; Sofia Beas et al., 2020; Li et al., 2022; Zhu et al., 2022). Subcortical afferents include both GABAergic and glutamatergic projections; however, beside a few examples (Zhang and van den Pol, 2017; Otis et al., 2019; Bruzsik et al., 2021; Zhu et al., 2022), it is presently unclear which subcortical input regions provide excitatory or inhibitory input to PVT.

The PVT also receives substantial cortical, glutamatergic inputs from the mPFC (Groenewegen, 1988; Sesack et al., 1989; Chen and Su, 1990; Hurley et al., 1991; Vertes, 2004; Li and Kirouac, 2012; Vertes et al., 2022) which are crucial for fear learning (Do-Monte et al., 2015), reward-related behaviors (Otis et al., 2019), and sociability (Yamamuro et al., 2020). Corticothalamic projections arise from layer 5 (L5) and layer 6 (L6) neurons. L5 axons typically drive strong thalamic excitation while L6 axons exert only modulatory control (Sherman and Guillery, 2005). mPFC has been shown to exert strong functional impact on PVT activity (Do-Monte et al., 2015; Otis et al., 2019; Aquino-Miranda et al., 2024). However, morphological studies indicate that PVT has very scarce “driver-like” L5 projections, most inputs were found to arise from L6 (Hurley et al., 1991; Li and Kirouac, 2012). L5 contacted PVT only indirectly via the GABAergic thalamic reticular nucleus (Aquino-Miranda et al., 2024; Ma et al., 2024). Thus, based on the present understanding of corticothalamic organization, the pure L6 inputs cannot explain the strong top-down cortical control of PVT.

Understanding the convergence or segregation of various cortical and subcortical inputs in the PVT requires an operational delineation of this nucleus. Immunohistochemical and gene expression studies have showed that in the PVT calretinin-positive (CR+) neurons form a spatially distinct, functionally coherent subpopulation that represent the major PVT cell type responsible for communication with the forebrain (Arai et al., 1994; Hua et al., 2018; Mátyás et al., 2018; Gao et al., 2023). PVT/CR+ cells provide the majority of the thalamic output to forebrain areas, are sensitive to various stressors, demonstrate a predictive increase in firing rate preceding behavioral arousal, and play an role in the manifestation of stress-induced alterations in behavior (Mátyás et al., 2018; Jász et al., 2025). Thus, here, we examined the precise spatial organization of the cortical and subcortical inputs relative to the distribution of PVT CR+ neurons. We employed vGAT-Cre and vGLUT2-Cre transgenic mice to map GABAergic and glutamatergic subcortical inputs to the PVT using both anterograde and retrograde viral tracing. We examined the laminar origin of cortical projections using layer-specific Cre-driver lines and CR immunostaining to define input distribution within the PVT. Our findings reveal a convergence of excitatory and inhibitory subcortical inputs with cortical L5 projections selectively in a CR-rich core region of PVT. In contrast, our data show that L6 projections predominantly innervate the surrounding transition zone.

## Materials and Methods

### Animals

In our experiments, we utilized adult (>3 months old) male and female transgenic mouse strains, including vGAT-iRES-FlpO (vesicular GABA transporter; B6.Cg-*Slc32a1^tm1.1(flpo)Hze^*/J; Stock No. 029591, RRID:IMSR_JAX:029591; *n* = 3 mice, *n*_male_ = 2, *n*_female_ = 1), vGAT-iRES-Cre (*Slc32a1^tm2(cre)Lowl^*/J; Stock No. 016962; RRID:IMSR_JAX:016962; *n* = 54 mice, *n*_male_ = 30, *n*_female_ = 24), vGluT2-iRES-Cre (vesicular glutamate transporter 2; Stock No. 016963; *Slc17a6^tm2(cre)Lowl^*/J; RRID:IMSR_JAX:016963; *n* = 34 mice, *n*_male_ = 20, *n*_female_ = 14), vGluT1-iRES-Cre (vesicular glutamate transporter 1; Stock No 023527; B6;129S-Slc17a7tm1.1(cre)Hze/J; RRID:IMSR_JAX:023527; *n* = 11, *n*_male_ = 3, *n*_female_ = 8), CR-iRES-Cre (calretinin, Stock No. 010774; B6(Cg)-*Calb2^tm1(cre)Zjh^*/J; RRID: IMSR_JAX:010774; *n* = 6 mice, *n*_male_ = 4, *n*_female_ = 2), Rbp4-Cre (retinol-binding protein 4; Stock No. 031125-UCD; Tg(Rbp4-cre)KL100Gsat/Mmcd; RRID:MMRRC_031125-UCD; *n* = 8 mice, *n*_male_ = 4, *n*_female_ = 4), Ntsr1-Cre (neurotensin receptor 1; Stock No. 030780-UCD; Tg(Ntsr1-cre)GN209Gsat/Mmucd; RRID:MMRRC_030780-UCD; *n* = 6 mice, *n*_male_ = 3 mice, *n*_female_ = 3), and FoxP2-Cre (forkhead box protein 2; Stock No. 030541; B6.Cg-Foxp^2tm1.1(cre)Rpa^/J; RRID:IMSR_JAX:030541; *n* = 3 mice, *n*_male_ = 2, *n*_female_ = 1). Additionally, we generated hybrid mice by crossbreeding vGAT-iRES-FlpO and vGluT2-iRES-Cre strains (*n* = 6 mice, *n*_male_ = 3, *n*_female_ = 3). In these strains, the Cre or Flp recombinase enzyme is coexpressed with the respective genes, enabling targeted genetic modifications. These strains were selected to facilitate precise genetic modifications in our study (Chaudhry et al., 1998; Takamori et al., 2001; Gong et al., 2007). We also used wild-type C57BL/6J mice (Jackson Laboratory; *n* = 5, *n*_male_ = 2, *n*_female_ = 3). The mice were housed in groups of 3–5 in a controlled environment with a 12 h dark/light cycle, constant temperature (22 ± 2°C), and humidity (60 ± 10%). Food and water were available *ad libitum*. All experimental procedures were approved by the Institutional Ethical Codex, Hungarian Act of Animal Care and Experimentation (1998, XXVIII, section 243/1998), and the Institutional Animal Care and Use Committee of the Institute of Experimental Medicine, Hungarian Research Network, Budapest and by the regulations of the European Union guidelines (directive 2010/63/EU). The experiments were performed by the National Animal Research Authorities of Hungary (PE/EA/877-7/2020).

### Stereotactic surgeries

Mice were deeply anesthetized with ketamine–xylazine (intraperitoneal injection, ketamine, 83 mg/kg; xylazine, 3.3 mg/kg). Depth of sleep was monitored throughout the surgery, if needed, supplemental dose (ketamine, 28 mg/kg; xylazine, 1.1 mg/kg) was injected intramuscularly. Externally applied lidocaine was used on the head and ears. The mice were placed in a stereotaxic frame (Kopf Instruments) on a heating pad to prevent hypothermia. We used eye protection gel against drying.

### Viral tracing

For viral tract tracing, we delivered Cre- or FlpO-dependent adeno-associated viral vectors (AAVs) expressing fluorescent proteins (EYFP, enhanced yellow fluorescent protein; EGFP, enhanced green fluorescent protein; or mCherry) to the relevant brain areas via stereotaxic surgery. To confirm the results, we tested two types of retrograde and several types of anterograde viral constructs in our experiments. For this, the animals were anesthetized with an intraperitoneal injection of ketamine–xylazine (83 mg/kg ketamine; 3.3 mg/kg xylazine) and then placed in a stereotaxic apparatus (Kopf Instruments). During surgery, additional anesthesia (28 mg/kg ketamine; 1.1 mg/kg xylazine) was administered intramuscularly if necessary. Eye gel was used to prevent eye drying, and lidocaine was applied to the head and ear areas for local anesthesia. The viral constructs were injected using a microinjector through a hole drilled in the skull, employing a glass capillary (20–100 nl, at a rate of 1 nl/s). The brain coordinates relative to bregma were determined based on the Paxinos mouse brain atlas (Paxinos and Franklin, 2001). The viral constructs used and the coordinates for virus injection are detailed in Tables 1 and 2.

**Table 1.** Viral constructs used for tract tracing experiments

Name	Produced by	Catalog #	Titer
AAV5-EF1a-double floxed-hChR2(H134R)-EYFP-WPRE-HGHpA	Addgene	20298	≥1 × 10^13^ vg/ml
AAV5-EF1a-double floxed-hChR2(H134R)-mCherry-WPRE-HGHpA	Addgene	20297	≥1 × 10^13^ vg/ml
AAVDJ-EF1a-fDIO-EYFP-WPRE	Addgene	55641	≥3 × 10^12^ vg/ml
AAV5-hSyn-DIO-mCherry	Addgene	50459	≥7 × 10^12^ vg/ml
AAV5-CAMKIIa-CHR2(H134R)-EYFP	Addgene	26969	≥1 × 10^13^ vg/ml
AAV5-EF1a-DIO-eYFP	Addgene	27056	≥1 × 10^13^ vg/ml
AAV5-EF1a-DIO-mCherry	UNC Vector Core	50462	≥7 × 10^12^ vg/ml
AAVrg-hSyn-DIO-EGFP	Addgene	50457	≥7 × 10^12^ vg/ml
AAVrg-hSyn-DIO-mCherry	Addgene	50459	≥7 × 10^12^ vg/ml

**Table 2.** Stereotaxic coordinates for viral injections

Brain region	AP	DV	ML
Bed nucleus of the stria terminalis (BNST)	−0.08	3.8	−0.8
Dorsomedial hypothalamic nucleus (DM)	−1.5	−5	−0.2
Lateral hypothalamus (LH)	−1.3	−5.15	−0.9
Lateral parabrachial nucleus (LPB)	−5.2	−3.4	−1.3
Medial prefrontal cortex (mPFC)	1.8	3	−0.4
Medial preoptic area (MPOA)	0.14	−5.5	−0.2
Parasubthalamic nucleus (PSTh)	−2.3	5.5	−1.2
Paraventricular thalamic nucleus (PVT)	−1.2	−2.9	0
Supramammillary nucleus (SUM)	−2.8	−4.8	0
Reticular thalamic nucleus (TRN)	−0.6	3.8	−1.2
Ventrolateral periaqueductal gray (vlPAG)	−4.6	−2.5	−0.5

### CTB+ retro-AAV tracing

For experiments in which a classical retrograde tracer was combined with a retrograde-transporting AAV, we injected a 1:1 (v/v) mixture of cholera toxin subunit B conjugated to Alexa Fluor 488 (CTB-Alexa 488; Thermo Fisher Scientific, catalog #C34775) and a retro-AAV vector (AAVrg-hSyn-DIO-mCherry, 7 × 10^12^ vg·ml). The solution was delivered stereotaxically (40 nl at a rate of 1 nl/s) in a glass capillary (tip *Ø* ≈ 25 µm) using the same coordinates and anesthesia regimen described above. To reduce back-flow, the capillary was left in place for 15 min after infusion before being withdrawn slowly. Animals then received routine postoperative care and survived for 21–30 d, allowing both robust retrograde transport of CTB-Alexa 488 and full expression of the viral reporter before perfusion–fixation.

### Tissue processing

Following the brain surgeries, ∼3 weeks were required for the viral proteins to express. After this period, the animals were transcardially perfused under deep anesthesia, during which the circulatory system was flushed first with 0.9% saline solution, followed by 120 ml of 4% paraformaldehyde (PFA) solution. The brains fixed in this manner were then removed from the skull, and after an additional 24 h of post-fixation (4% PFA), 50-μm-thick coronal brain sections were prepared using a vibratome (Leica).

### Fluorescent immunohistochemistry

To amplify the viral fluorescent signal, we performed fluorescent immunostaining with anti-GFP/anti-RFP (green and red fluorescent protein) antibodies against the reporter proteins expressed by the viruses (EGFP, EYFP, mCherry; Biro et al., 2018; Bruzsik et al., 2021; Babiczky and Matyas, 2022; Jász et al., 2025). Additionally, to facilitate the identification of nucleus boundaries, we used antibodies against various marker proteins (calretinin, calbindin, parvalbumin, serotonin, choline acetyltransferase; for details see Table 3). The CTB-Alexa 488 signal was further amplified by incubating sections with a goat anti-CTB primary antibody (Table 3), followed by the corresponding fluorophore-conjugated secondary antibody (Table 4).

**Table 3.** Overview of primary antibodies used in this study

Antigen targeted	Host	Dilution	Catalog number	Source	RRID
GFP	Chicken	1:1,000	A10262	Thermo Fisher Scientific	AB_2534023
RFP	Rabbit	1:1,000	600-401-379	Rockland Immunochemicals	AB_2209751
CR	Guinea pig	1:500	214-104	Synaptic Systems	AB_10635160
CR	Mouse	1:500	6B3	Swant	AB_10000320
CTB	Goat	1:2,000	703	List Biological Laboratories	AB_10013220
PV	Guinea pig	1:1,000	195-004	Synaptic Systems	AB_2156476
PV	Mouse	1:1,000	P3088	Sigma-Aldrich	AB_477329
5-HT	Goat	1:8,000	20079	ImmunoStar	AB_572262
ChAT	Rabbit	1:2,000	297-013	Synaptic Systems	AB_2620040
CB	Guinea pig	1:2,000	214-004	Synaptic Systems	AB_10550535

**Table 4.** Overview of secondary antibodies used in this study

Antigen targeted	Host	Fluorescent dye	Dilution	Catalog number	Source	RRID
Chicken	Donkey	Alexa Fluor 488 AffiniPure	1:500	703-545-155	Jackson ImmunoResearch	AB_2340375
Goat	Donkey	Alexa Fluor 488 AffiniPure	1:500	705-545-147	Jackson ImmunoResearch	AB_2336933
Rabbit	Donkey	Cy3 AffiniPure	1:500	711-165-152	Jackson ImmunoResearch	AB_2307443
Guinea pig	Donkey	Alexa Fluor 647 AffiniPure	1:500	706-605-148	Jackson ImmunoResearch	AB_2340476
Mouse	Donkey	Alexa Fluor 647 AffiniPure	1:500	715-605-151	Jackson ImmunoResearch	AB_2340863
Mouse	Donkey	Alexa Fluor 488 AffiniPure	1:500	715-545-151	Jackson ImmunoResearch	AB_2341099
Goat	Donkey	Cy5 AffiniPure	1:500	705-175-147	Jackson ImmunoResearch	AB_2340415
Guinea pig	Donkey	Alexa Fluor 647 AffiniPure	1:500	706-605-148	Jackson ImmunoResearch	AB_2340476

After washing with 0.1 M PB (phosphate buffer), the free-floating sections were treated for 2 h with a mixture of 10% normal donkey serum and 2% Triton-X-100 dissolved in PB, to block endogenous nonspecific binding sites and permeabilize the membranes as described previously (Biro et al., 2017, 2023; Jász et al., 2025). The sections were then incubated for 24 h in a mixture of 2% normal donkey serum and primary antibodies dissolved in 0.1 M PB. After another washing step, the sections were treated for 2 h with fluorescently labeled secondary antibodies and a nuclear stain (1:2,000, Hoechst 33258, Sigma-Aldrich). Finally, after washing, the sections were mounted on slides in a chromium gelatin solution and covered with Mowiol 4-88 (81381, Sigma-Aldrich). The primary and secondary antibodies used are detailed in Tables 3 and 4.

### Fluorescent and confocal microscopy

The slides were scanned using a Pannoramic Digital Slide Scanner (Pannoramic MIDI II, 3DHISTECH, Budapest) with a Zeiss Plan-Apochromat 10× objective [numerical aperture (NA): 0.45; *x*–*y* resolution: 0.65 μm/pixel], and the microscopic images were analyzed using CaseViewer 2.3 (3DHISTECH) software (Bruzsik et al., 2021; Bősz et al., 2025; Jász et al., 2025). To achieve higher optical resolution and contrast, we often use confocal microscopy, which filters out out-of-focus light during the imaging process. For the acquisition of representative images from the sections, we used a Nikon C2 confocal microscope (Nikon Europe) with 488, 561, and 642 nm lasers (CVI Melles Griot). Imaging was performed with a 4× Plan Fluor objective (NA: 0.13; *x–y*: 2 μm/pixel), a 10× Plan Fluor objective (NA: 0.13; *x–y*: 0.63 μm/pixel; *z*-step size 2 μm), and a 20× CFI Plan Apo VC objective (NA: 0.75; *x–y*: 0.32 μm/pixel; *z*-step size 1 μm, Nikon Europe). Image intensity and contrast adjustments were made using FIJI ImageJ 1.52a image analysis software.

For more detailed analysis and to examine fluorescent fibers in the cortical and subcortical brain regions within the PVT, we used a Nikon A1R confocal microscope (Nikon Europe) with 488, 561, and 642 nm lasers (CVI Melles Griot). The scanning was done in line series mode. Since confocal imaging is time consuming, we selected the middle part of the PVT for axon density quantification, the same area targeted in retrograde virus injection experiments. Images were acquired from a single section per animal, and a 4 × 5 montage was assembled at 60× magnification (Nikon CFI Plan Apo VC 60×, NA: 1.40 oil objective) to fully capture axon branching in the MD and PVT nuclei. Both structures were then systematically sampled along the *y* and *x* axes at 100 μm intervals, using windows of 89.51 μm × 89.51 μm with an in-plane resolution of 0.09 μm/pixel. This resulted in 18–24 *z*-stacks depending on the section and the extent of the nuclei. The *z*-stacks contained 57 slices with a *z*-step size of 0.09 μm/pixel, covering a total of 5 μm along the *z*-axis. This generated isotropic voxels, meaning the spatial resolution was uniform both in-plane and along the *z*-axis.

### Quantification of retrogradely labeled cells using viral tracing

The location of retrogradely labeled cells was determined by comparing the sections with the Paxinos brain atlas (Paxinos and Franklin, 2001) and using background staining. The labeled cells were then marked in the atlas using Adobe Photoshop software. The quantitative distribution of retrogradely labeled cells between different subcortical brain regions was determined manually. To establish the ratios, the absolute number of cells found in each region was normalized to the total number of subcortical cells found in the entire brain (cells not identified as PVT inputs, based on control experiments and possibly due to viral spread to adjacent structures, were not included in the ratio calculations).

### Axon density analysis

We included all *z*-stacks from the selected section that did not show damage due to tearing, ensuring coverage of different and representative areas of both nuclei (PVT and MD), including denser and sparser axon networks. The resulting *z*-stacks were analyzed using FIJI ImageJ 1.52a software (Schneider et al., 2012), which includes a variety of built-in modules and supports the use of custom scripts, facilitating scientific image analysis. The software was used to measure axon density in cortical and subcortical brain areas for each recorded image. The custom code used for axon quantification in this study is available at https://github.com/lorinczszabolcs/axon-quantification.

For the analysis of axon density, we used all previously selected *z*-stacks, performing the analysis through an automated procedure. For this, we developed a FIJI ImageJ script that included several steps. The first step was the automatic identification and removal of boutons from the *z*-stacks. We then identified the axons, located their midlines, and calculated their total length. The following paragraphs detail the steps of the process.

To identify boutons, we used the FIJI ImageJ Trainable Weka Segmentation (Arganda-Carreras et al., 2017) plugin, which applies a random forest machine learning model. The input to the model was a series of *z*-stacks, which included the original *z*-stack and various augmentations, such as features computed by FeatureJ, including the eigenvalues of the Hessian matrix, higher-order derivatives of the input data, the Laplacian, as well as the difference of Gaussians and the mean and variance of the neighborhood of each voxel. The trained models were then used for batch processing the remaining *z*-stacks, generating segmentation maps. In these, the voxels representing boutons were set to zero, while the rest of the voxels were set to one. The intensity values of the original *z*-stack were then multiplied by the segmentation maps, resulting in bouton-free *z*-stacks.

After removing the boutons, we performed morphological analysis to identify the axons. First, we computed the “tubeness” feature (Sato et al., 1998), which evaluated each voxel based on the likelihood of tube-like structures (axons) being present. We utilized a Gaussian filter with a standard deviation (*σ*) of 0.32 μm. This *σ* value was derived from the average diameter of several manually measured axons, allowing us to detect axons with a minimum diameter of 0.32 μm. We then applied bilateral thresholding, and the resulting binary mask was used to extract the midlines of the axons using the Skeletonize3D plugin (Arganda-Carreras et al., 2010). Finally, we calculated the total length of the axons in the given *z*-stack and determined the axon density as the ratio of total axon length to the volume of the *z*-stack.

### Statistical analysis

Statistical analyses were conducted using GraphPad Prism version 9.5.1 (GraphPad Software). One-way ANOVA or one-way repeated-measures ANOVA, followed by Tukey's post hoc test, was used to compare axonal densities across regions. Data from the retrograde cell labeling experiments were not subjected to statistical analysis. To examine the relationship between axon density and CR fluorescence intensity, we performed Pearson’s correlation analysis. The significance threshold was set at *p* < 0.05, and results are reported as mean ± standard error of the mean (SEM).

## Results

### Retrograde tracings, injection sites, and delineation of PVT

**Figure 1.**
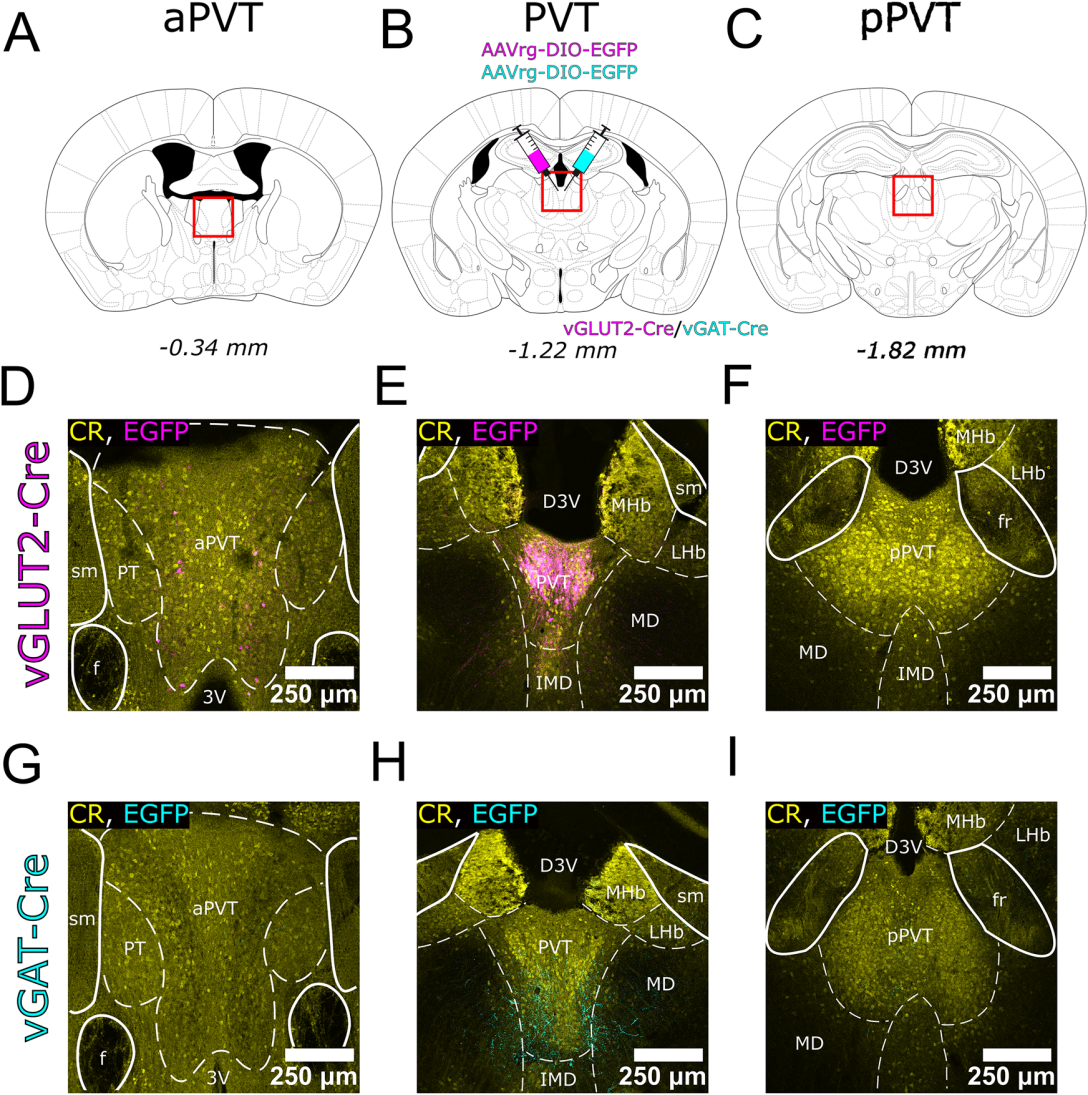
Representative examples of retrograde virus injection sites in the PVT. ***A–C***, Three coronal planes according to the Paxinos Atlas at the anterior, middle, and posterior levels of the paraventricular thalamic nucleus (PVT), with numbers indicating the distance from the bregma. Injections were performed at the middle coronal level. ***D–F***, AAVrg virus expression in PVT neurons at the injection site shown in the three planes of a representative vGLUT2-Cre animal. The sections are immunostained for calretinin to label PVT. ***G–I***, The same as in ***D–F*** in a representative vGAT-Cre animal. Note that in this case the extent of the injection site is visible via the dendritic arbors of the scattered interneurons. 3V, third ventricle; AAVrg, retrograde adeno-associated virus; aPVT, anterior PVT; CR, calretinin; D3V, dorsal part of the third ventricle; EGFP, enhanced green fluorescent protein; f, fornix; fr, fasciculus retroflexus; IMD, intermediodorsal nucleus; MD, mediodorsal nucleus; MHb, medial habenula; LHb, lateral habenula; pPVT, posterior PVT; PT, paratenial nucleus; sm, stria medullaris thalami; vGAT, vesicular GABA transporter; vGLUT2, vesicular glutamate transporter 2.

First, we aimed to determine the sources of subcortical glutamatergic (excitatory) and GABAergic (inhibitory) inputs to the PVT by injecting retrograde viruses (AAVrg-GFP) into the PVT of vesicular glutamate transporter type 2-Cre (vGLUT2-Cre) and vesicular GABA transporter-Cre (vGAT-Cre) transgenic mouse strains (Fig. 1*A–C*). The PVT is frequently categorized into distinct subregions, primarily the anterior paraventricular thalamus (aPVT) and posterior paraventricular thalamus (pPVT). These subdivisions exhibit divergent input and projection characteristics as well as distinct functional roles, as evidenced by numerous studies (Moga et al., 1995; Li and Kirouac, 2012; Gao et al., 2020). In the retrograde tracing experiments, we targeted the middle part of the nucleus (from the bregma −0.90 to −1.50 mm; Fig. 1) that recently has been shown to actively participate in the stress-induced alteration of spontaneous behaviors (Jász et al., 2025). None of the injections invaded the anterior PVT (Fig. 1*D*,*G*), though scattered neuropil labeling was observed in vGAT-Cre injections (Fig. 1*H*), and a few cells were present in vGLUT2-Cre mice (Fig. 1*E*).

Given that a majority of PVT neurons exhibit CR expression (Arai et al., 1994; Mátyás et al., 2018; Gao et al., 2023), we employed CR immunostaining to both anatomically delineate the boundaries of the PVT and trace the spread of injections in each retrograde experiment relative to neighboring structures such as the mediodorsal nucleus (MD), lateral habenula (LHb), and intermediodorsal thalamic nucleus (IMD; Fig. 1, Fig. S1).

In total, we performed 10 injections in vGLUT2-Cre and 21 in vGAT-Cre transgenic mouse strains, from which we selected those three cases of each strain for quantitative analysis, in which the injection sites were restricted to PVT with minimal or no spread to the neighboring nuclei (Fig. 1) All injection sites were referenced to CR immunostaining. We used *n* = 3 vGLUT2-Cre and *n* = 6 vGAT-Cre mice with injections to the neighboring territories as controls. In these animals the injection sites mainly involved MD or LHb. The remaining animals (*n* = 4 vGLUT2-Cre and *n* = 12 vGAT-Cre), in which the injection sited involved PVT but spread to neighboring areas were used to confirm the observations obtained with the selective injections.

The extent of the injection sites could be clearly identified in the case of the vGLUT2-Cre animals since thalamocortical cells in PVT express vGLUT2 (Fig. 1). In the case of vGAT-Cre animals, the sparse labeling of vGAT+ interneurons (Arcelli et al., 1997) in PVT allowed us to localize the spread of the injection sites (Fig. 1).

In vGLUT2-Cre mice used for quantitative analysis, AAVrg-GFP injections were placed within the middle of the PVT (Fig. 1). Specifically, Case #LB83 was confined within the PVT, whereas in Case #LB89 and #LB90, viral spread slightly affected the transition zone between the MDm and PVT.

In the case of vGAT-Cre experiments, AAVrg-GFP injections were placed within the middle of the PVT (Fig. 1). Specifically, Case #LB20 showed a confined deposition within the PVT, whereas in Case #LB17 and #LB145, viral spread minimally affected the medial sector of the MD and the dorsal portion of the IMD.

Our subsequent anterograde tracing experiments (see below) confirmed PVT afferents from all input regions identified with the retrograde tracings. This also shows that our retrograde injection sites represent well the input space of the PVT.

### Identification of subcortical inputs to the PVT

**Figure 2.**
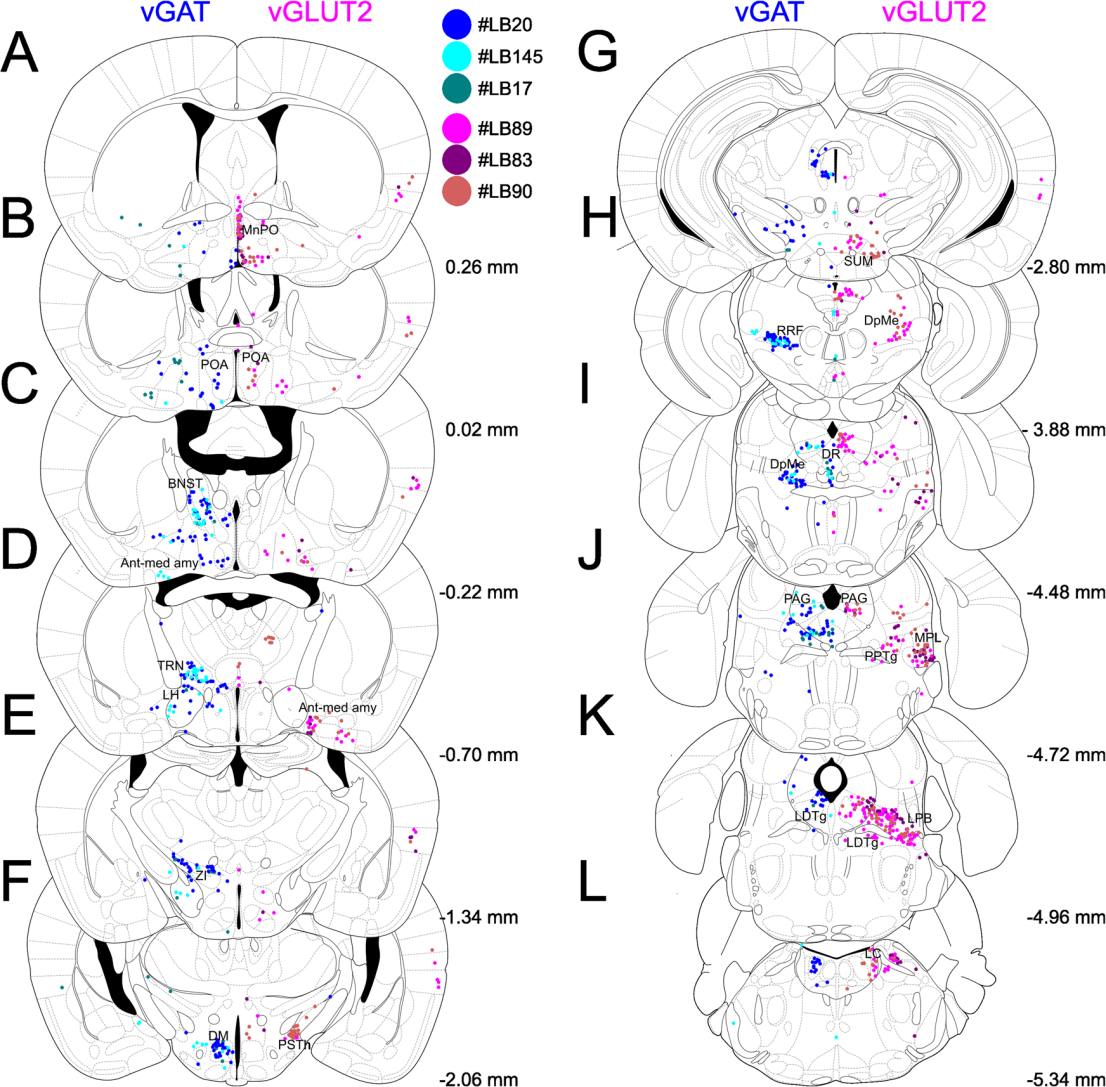
Distribution of retrogradely labeled vGAT and vGLUT2 cells from the PVT using viral tracing. ***A–L***, The locations of retrogradely labeled cells in coronal planes from anterior to posterior in vGAT-Cre (*n* = 3) and vGLUT2-Cre (*n* = 3) animals, with numbers indicating the distance from the bregma. A dot represents a neuron. Different animals are represented by different hues of blue (vGAT) or magenta (vGLUT2). Note the difference in the location of excitatory and inhibitory inputs and the consistency of the labeling among the animals of the same strains. Ant-med amy, anterior and medial amygdaloid region; BNST, bed nucleus of the stria terminalis; DM, dorsomedial hypothalamic nucleus; DpMe, deep mesencephalic area; DR, dorsal raphe; LC, locus ceruleus; LDTg, laterodorsal tegmental nucleus; LH, lateral hypothalamus; LPB, lateral parabrachial nucleus; MnPO, median preoptic nucleus; MPL, medial paralemniscal nucleus; PAG, periaqueductal gray; POA, preoptic area; PPTg, pedunculopontine tegmental nucleus; PSTh, parasubthalamic nucleus; RRF, retrorubral area; SUM, supramammillary nucleus; TRN, reticular thalamic nucleus; vGAT, vesicular GABA transporter; vGLUT2, vesicular glutamate transporter 2; ZI, zona incerta.

In agreement with the previous track tracing studies (Cornwall and Phillipson, 1988b; Krout and Loewy, 2000a,b; Li and Kirouac, 2008), we identified high number of subcortical cells projecting to the PVT across numerous brain regions, spanning nearly the entire anteroposterior axis from the preoptic area (POA) and bed nucleus of the stria terminalis (BNST) to the parabrachial nucleus (LPB) and locus ceruleus (LC; Fig. 2). The brains have not been analyzed more caudally. Most of the identified input regions are corroborated by the existing literature (Marchant et al., 2010; Li and Kirouac, 2012; Vertes et al., 2022; Kirouac, 2025). Our current approach, however, provided information on the excitatory versus inhibitory nature of these inputs.

**Figure 3.**
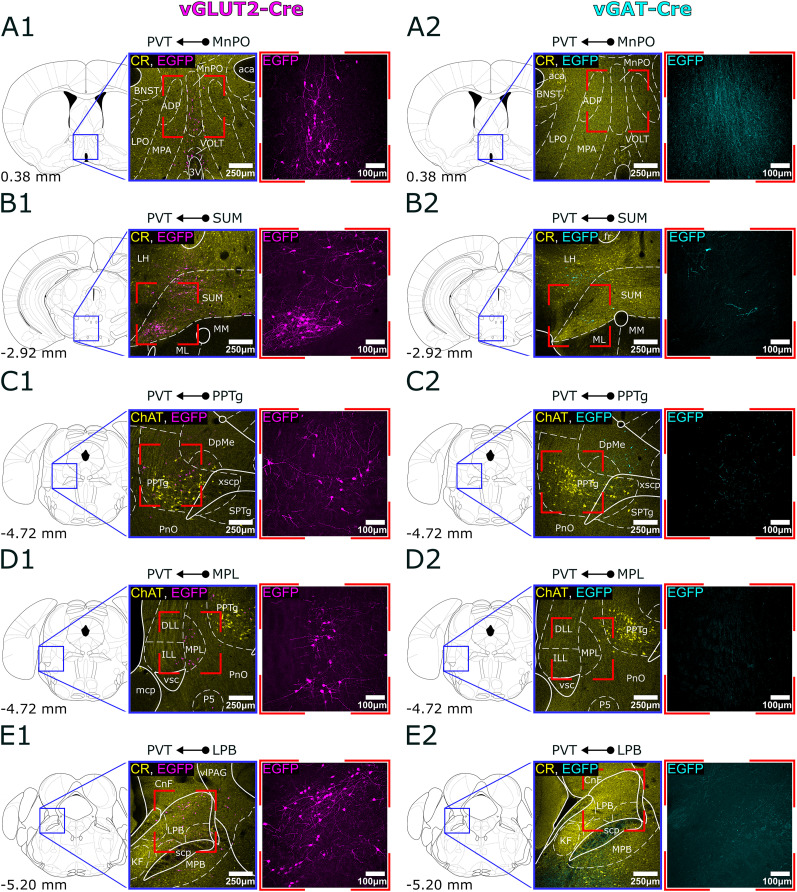
Glutamatergic, subcortical input regions of PVT. ***A1–E1***, Representative confocal images of retrogradely labeled cells in a vGLUT2-Cre animal. The locations of each input region (MnPO, SUM, PPTg, MPL, LPB) from anterior to posterior are shown in the stereotaxic brain atlas, along with images of the labeled cells at 10× and 20× magnification in vGLUT2-Cre animals. ***A2–E2***, Absence of labeled cells in the same regions in a vGAT-Cre animal. To facilitate nuclear localization the low power images in ***A1***,***2***, ***B1***,***2***, and ***E1***,***2*** are immunostained for CR, whereas in ***C1*–*D2*** for ChAT. 3V, 3rd ventricle; aca, anterior commissure anterior part; ADP, anterodorsal preoptic nucleus; BNST, bed nucleus of the stria terminalis; ChAT, choline acetyltransferase; CnF, cuneiform nucleus; CR, calretinin; DLL, dorsal nucleus of the lateral lemniscus; DpMe, deep mesencephalic area; EGFP, enhanced green fluorescent protein; fr, fasciculus retroflexus; ILL, intermediate nucleus of the lateral lemniscus; KF, Kölliker–Fuse nucleus; MPB, medial parabrachial nucleus; LH, lateral hypothalamus; LPB, lateral parabrachial nucleus; LPO, lateral preoptic area; mcp, middle cerebellar peduncle; ML, medial part of the mammillary body; MM, medial part of the mammillary body; MnPO, median preoptic nucleus; MPA, medial preoptic area; P5, peritrigeminal zone; PnO, oral part of the pontine reticular nucleus; PPTg, pedunculopontine tegmental nucleus; PVT, paraventricular thalamic nucleus; scp, superior cerebellar peduncle; SPTg, subpeduncular tegmental nucleus; SUM, supramammillary nucleus; vGAT, vesicular GABA transporter; vGLUT2, vesicular glutamate transporter 2; vlPAG, ventrolateral periaqueductal gray; VOLT, vascular organ of the lamina terminalis; vsc, ventral spinocerebellar tract; xscp, crossing of the superior cerebellar peduncle.

**Figure 4.**
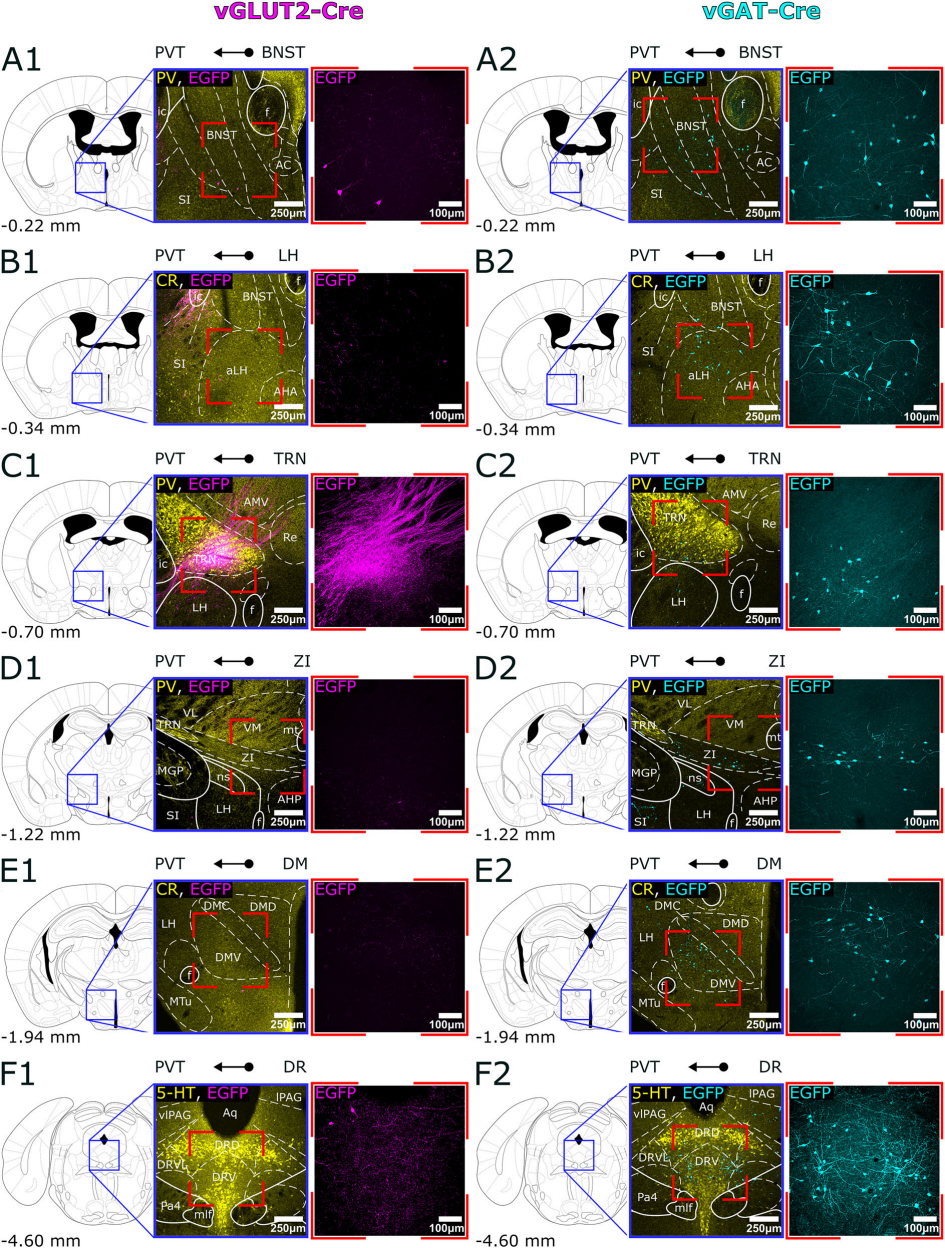
GABAergic, subcortical input regions of PVT. ***A1–F1***, Absence of retrogradely labeled cells in the GABAergic input regions of PVT (BNST, LH, TRN, ZI, DM, DR) in a vGLUT2-Cre animal shown from anterior to posterior in the stereotaxic brain atlas, along with representative images at 10× and 20× magnification. The labeling is ***C1*** represents the vGLUT2+ axons of PVT in the TRN. ***A2–F2***, Retrogradely labeled cells from the PVT in the same regions in a vGAT-Cre animal. To facilitate nuclear localization the low power images of ***A1***,***2***, ***C1***,***2***, and ***D1***,***2*** are immunostained for PV, in ***B1***,***2*** and ***E1***,***2***, for CR, and in ***F1***,***2*** for 5-HT. 5-HT, serotonin; AC, anterior commissure nucleus; AHA, anterior hypothalamic nucleus; anterior part; AHP, anterior hypothalamic nucleus; posterior part; aLH, anterior LH; AMV, anteromedial thalamic nucleus; ventral part; Aq, aqueduct of the brain; BNST, bed nucleus of the stria terminalis; CR, calretinin; DM, dorsomedial hypothalamic nucleus; DMD, DM dorsal part; DMC, DM central part; DMV, DM ventral part; DR, dorsal raphe; DRV, DR ventral part; DRVL, DR ventrolateral part; EGFP, enhanced green fluorescent protein; f, fornix; ic, internal capsule; LH, lateral hypothalamus; lPAG, lateral periaqueductal gray; MGP, medial globus pallidus; mt, mammillothalamic tract; mlf, medial longitudinal fasciculus; MTu, medial tuberal nucleus; ns, nigrostriatal bundle; Pa4, paratrochlear nucleus; PV, parvalbumin; PVT, paraventricular thalamic nucleus; Re, reuniens nucleus; SI, substantia innominata; TRN, reticular thalamic nucleus; vGAT, vesicular GABA transporter; vGLUT2, vesicular glutamate transporter 2; VM, ventromedial thalamic nucleus; VL, ventrolateral thalamic nucleus; vlPAG, ventrolateral periaqueductal gray; ZI, zona incerta.

**Figure 5.**
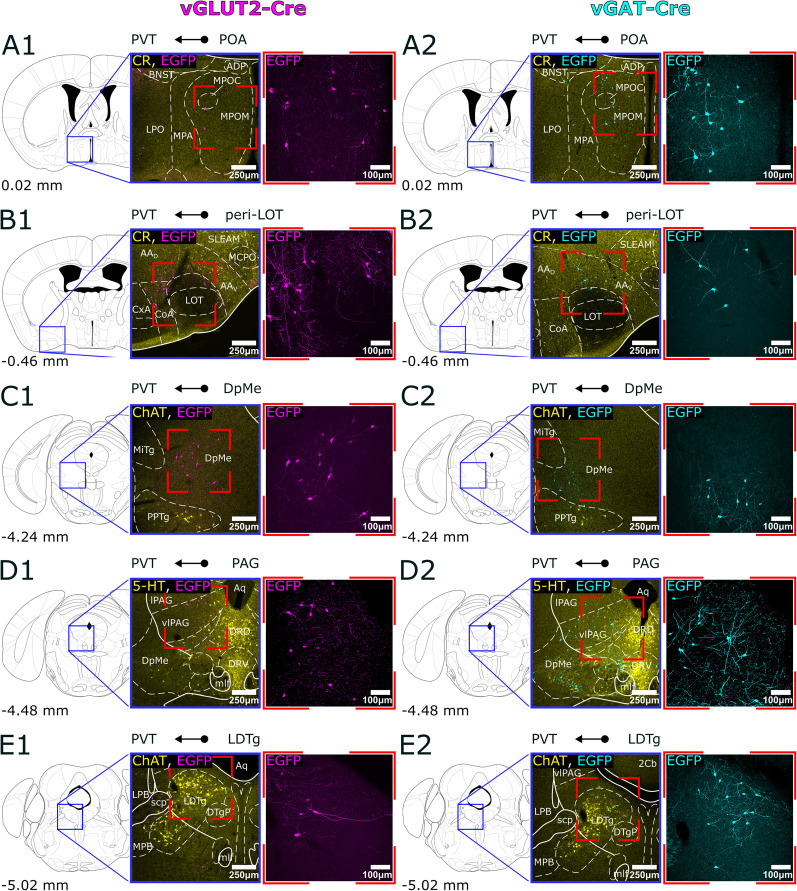
Mixed, glutamatergic and GABAegic, subcortical input regions of the PVT. ***A1–E1***, Representative confocal images of retrogradely labeled cells from the PVT in a vGLUT2-Cre animal. The locations of each input region (POA, peri-LOT, DpMe, PAG, LDTg) from anterior to posterior in the stereotaxic brain atlas, along with images of labeled cells at 10× and 20× magnification in vGLUT2-Cre animals. ***A2–E2***, Representative confocal images of retrogradely labeled cells in a vGAT-Cre animal in the same regions. To facilitate nuclear localization, the low power images in ***A1***,***2*** and ***B1***,**2** are immunostained for CR, ***C1***,***2***, in ***E1***,***2*** for ChAT, and in ***D1***,***2*** for 5-HT. 5-HT, serotonin; AAD, dorsal anterior amygdaloid region; AAV, ventral anterior amygdaloid region; ACo, anterior cortical amygdaloid nucleus; ADP, anterodorsal preoptic nucleus; Aq, aqueduct of the brain; BNST, bed nucleus of the stria terminalis; ChAT, choline acetyltransferase; CR, calretinin; CxA, cortex-amygdala transition zone; DpMe, deep mesencephalic area; DRD, dorsal part of the dorsal raphe; DRV, ventral part of the dorsal raphe; DTgP, dorsal tegmental nucleus; pericentral part; EGFP, enhanced green fluorescent protein; LOT, lateral olfactory tract nucleus; lPAG, lateral periaqueductal gray; MCPO, magnocellular preoptic nucleus; MiTg, microcellular tegmental nucleus; mlf, medial longitudinal fasciculus; MPA, medial preoptic area; MPB, medial parabrachial nucleus; MPOC, central part of the medial preoptic nucleus; MPOM, medial part of the medial preoptic nucleus; LDTg, laterodorsal tegmental nucleus; LPB, lateral parabrachial nucleus; LPO, lateral preoptic area; POA, preoptic area; PPTg, pedunculopontine tegmental nucleus; PVT, paraventricular thalamic nucleus; scp, superior cerebellar peduncle; SLEAM, medial part of the sublenticular extended amygdala; vGAT, vesicular GABA transporter; vGLUT2, vesicular glutamate transporter 2; vlPAG, ventrolateral periaqueductal gray.

By comparing the distribution of retrogradely labeled cells in vGAT-Cre and vGLUT2-Cre mice, we categorized the subcortical input regions into three groups: those providing exclusively glutamatergic innervation, those providing exclusively GABAergic innervation, and those containing both types of cells (Figs. 3–5).

We also observed scattered retrogradely labeled cells in cortical regions, including the prelimbic and insular cortices, in both vGAT-Cre and vGLUT2-Cre animals. These cortical areas have known inputs to the PVT (Li and Kirouac, 2012), and their labeling in our experiments likely reflect low-level Cre expression.

**Figure 6.**
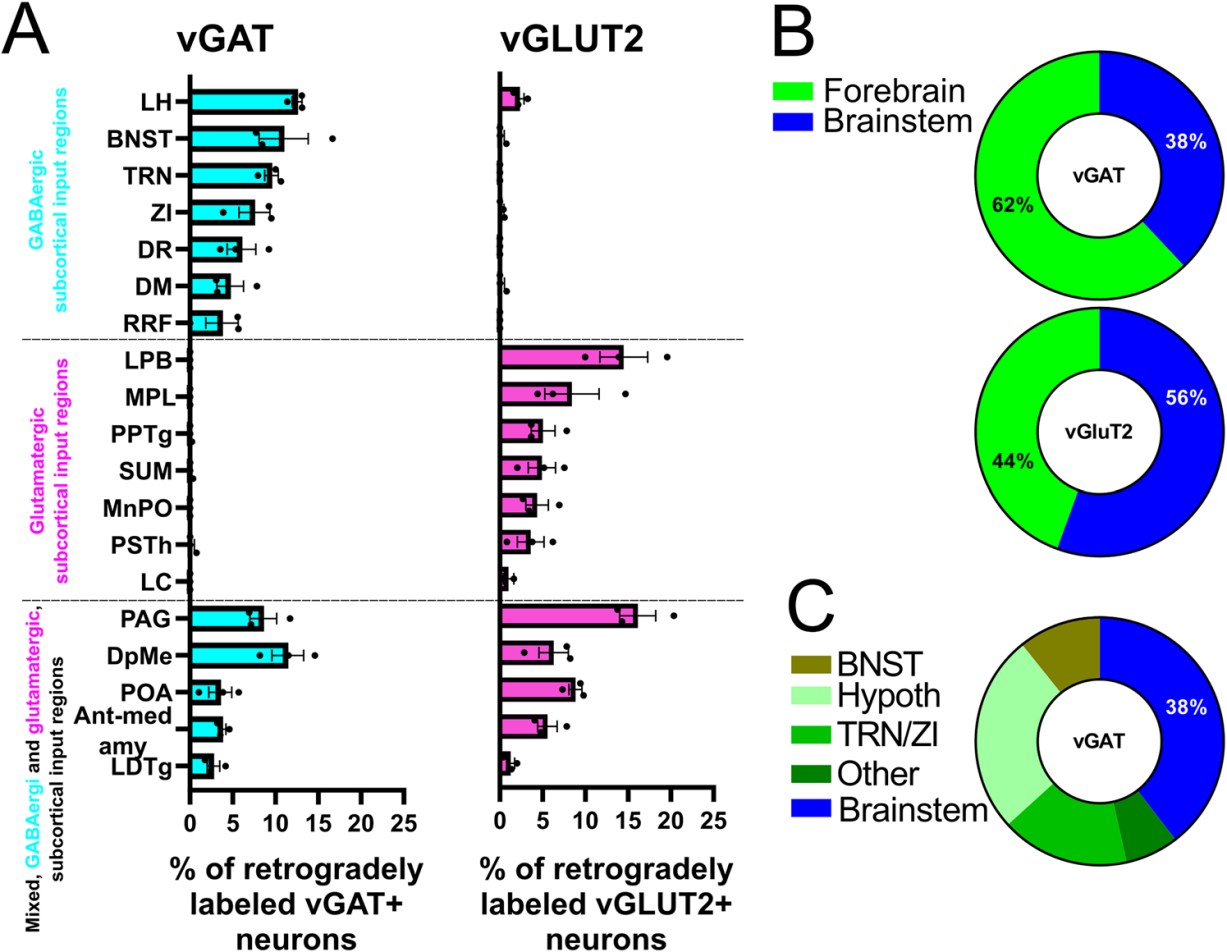
Quantitative analysis of the number of retrogradely labeled cells. ***A***, The proportion of retrogradely labeled cells found in each input region relative to the total number of subcortical labeled cells found in vGAT-Cre mice (*n* = 3, life) and in vGLUT2-Cre (*n* = 3) mice. The brain regions are grouped according to phenotype of the inputs; GABAergic (top), glutamatergic (middle) and mixed (bottom). ***B***, Pie charts illustrating the proportion of total vGAT and vGLUT2 inputs originating from the forebrain and brainstem. ***C***, Breakdown of vGAT inputs within the forebrain, showing the relative contribution of different forebrain regions. Percentages indicate the relative contribution of each region to the total input. The data are presented as mean ± standard error. Ant-med amy, anterior and medial amygdaloid region; BNST, bed nucleus of the stria terminalis; DM, dorsomedial hypothalamic nucleus; DpMe, deep mesencephalic area; DR, dorsal raphe; LC, locus ceruleus; LDTg, laterodorsal tegmental nucleus; LH, lateral hypothalamus; LPB, lateral parabrachial nucleus; MnPO, median preoptic nucleus; MPL, medial paralemniscal nucleus; PAG, periaqueductal gray; POA, preoptic area; PPTg, pedunculopontine tegmental nucleus; PSTh, parasubthalamic nucleus; RRF, retrorubral area; SUM, supramammillary nucleus; TRN, reticular thalamic nucleus; vGAT, vesicular GABA transporter; vGLUT2, vesicular glutamate transporter 2; ZI, zona incerta.

To quantitatively assess the proportion of GABAergic and glutamatergic input neurons throughout the entire brain, we counted labeled neurons along the neuro-axis in vGAT-Cre (*n* = 3) and vGLUT2-Cre mice (*n* = 3). We normalized the number of neurons found in the specific nucleus to the total number of subcortical cells identified throughout the brain (∼180–880 cells per mouse) in both vGAT-Cre (mean ± SEM: 469.3 ± 210.3 cells) and vGLUT2-Cre (mean ± SEM: 476 ± 167.6 cells; Fig. 6). In the description below, we describe only those brain regions that contained >3% of the retrogradely labeled cells (altogether 77.75% vGLUT2 and 78.63% of the vGAT cells). The remaining cells (22.25% vGLUT2 and 21.37% of the vGAT cells) were scattered throughout the hypothalamus and the brainstem.

In order to assign a given brain region to vGLUT2+, vGAT+, or mixed, we determined the ratio of retrogradely labeled vGAT+ and vGLUT2+ and neurons. If this ratio was below 0.2 (i.e., 5 times more vGAT+ cells), we considered the region vGAT+, if this ratio was between 0.2 and 5, we defined the region as mixed and above 5 (i.e., 5 times more vGLUT2 cells) as vGLUT2+.

### Subcortical brain regions with only glutamatergic (vGLUT2) PVT-projecting neurons

Following viral injections into the PVT of vGLUT2-Cre mice, 71.57% of the retrogradely labeled cells were confined to brain regions that provided only vGLUT2+ innervation to PVT. In the hypothalamus we found a dense population of glutamatergic neurons in the median preoptic nucleus (MnPO) located at the level of the anterior commissure and slightly anterior to it (Figs. 2*A*, 3*A1*; 4.35% ± 1.31% of all vGLUT2+ cells). Similarly, a strong retrogradely labeled cell population was visualized in the caudal region of the hypothalamus within the supramammillary nucleus (SUM, 4.92% ± 1.60%; Figs. 2*G*, 3*B1*), where both the medial and lateral parts contained neurons projecting to the PVT. The third significant glutamatergic hypothalamic region identified was the parasubthalamic nucleus (PSTh, 3.60% ± 1.50%; Fig. 2*F*), which, despite its small size, exhibited a dense population of projecting neurons. In vGAT-Cre animals, no retrogradely labeled cells were observed in these regions (Fig. 3*A2*,*B2*), suggesting that the MnPO, SUM, and PSTh primarily provide excitatory innervation to the PVT.

Within the brainstem we identified PVT-projecting cells in the vGLUT2-Cre animals, in the pedunculopontine tegmental nucleus (PPTg, 5.1% ± 1.39%) and the medial paralemniscal nucleus (MPL, 8.44% ± 3.17%). The largest population of pure vGLUT2+ neurons projecting to PVT was found in the LPB (14.49% ± 2.79%). In addition, we found scattered neurons within and around the LC (Figs. 2*J–L*, 3*C1*–*E1*). No labeled cells were found in these nuclei in the vGAT-Cre animals (Fig. 3*C2*–*E2*). The PPTg is a cholinergic nucleus, and we used immunostaining against choline acetyltransferase (ChAT) to delineate its boundaries. The cholinergic cells labeled by immunostaining did not show colocalization with the retrogradely labeled vGLUT2+ cells, which is consistent with the previous literature (Wang and Morales, 2009; Luquin et al., 2018).

Our data shows that the LPB is the most significant PVT-projecting, purely glutamatergic population from the brainstem whereas the hypothalamus contains small but still significant projections in its most rostral and caudal regions (Fig. 6*A*,*B*).

### Subcortical brain regions with only GABAergic (vGAT) PVT-projecting neurons

Following viral injections into the PVT of vGAT-Cre mice, 69.91% of the retrogradely labeled cells were confined to brain regions that provided only vGAT2+ innervation to PVT. In the forebrain, the highest number of retrogradely labeled GABAergic cells were observed in the BNST (10.95% ± 2.87%) following vGAT-Cre injections to PVT (Figs. 2*C*, 4*A1*). No neuron was labeled in this nucleus in the vGLUT2-Cre animals (Fig. 4*A2*). The identified population was located predominantly in the posterior part of the nucleus, particularly within the posteromedial, posterointermediate, and posterolateral sectors, situated between the substantia innominata (SI) and the fornix, at the level of the optic chiasm. Additionally, we found a few scattered neurons in the basal forebrain [specifically in the ventral pallidum (VP), SI, and the vertical and horizontal branches of Broca's diagonal bundle]; however, this is likely a consequence of the virus leaking into the medial dorsal thalamus (MD), as suggested by control experiments and literature (Groenewegen et al., 1993; Zahm et al., 1996).

In the hypothalamus, the largest input region identified in vGAT-Cre animals was the lateral hypothalamus (LH, 12.53% ± 0.57%), where a dense population was observed, particularly in the anterior regions, although scattered cells were found throughout the nucleus's entire anteroposterior extent (Figs. 2*D*, 4*B1*,*2*). We also found a few cells in the LH of the vGLUT2-Cre mice, consistent with an earlier physiological study (Otis et al., 2019), but since their number was very low (vGAT+/vGLUT2+ ratio 0.18), we classified the LH a GABAergic input region. Another major GABAergic input region from the hypothalamus nucleus was identified as the dorsomedial nucleus (DM, 4.70% ± 1.57%; Figs. 2*F*, 4*E1*,*2*), while the suprachiasmatic nucleus (SCh) contained only sporadic cells.

In the rest of the diencephalon, we identified GABAergic inputs to PVT from the ventromedial sector of the anterior thalamic reticular nucleus (TRN, 9.53% ± 0.81%) marked by PV immunostaining (Pinault, 2004; Hádinger et al., 2023) and the anterior portion of the zona incerta (ZI, 7.54% ± 1.82%; Figs. 2*D*,*E*, 4*C1*,*D1*,*C2*,*D2*).

In the brainstem, retrogradely labeled cells were found only in the dorsal raphe (DR, 6.04% ± 1.68%) region and the retrorubral area following vGAT-Cre injections (Figs. 2*H*,*I* 4*F1*,*2*). We employed immunostaining against serotonin (5-HT) for the identification of the dorsal raphe, where the labeled cells were predominantly located in the lateral sections. Similar to the PPTg, the serotonergic and retrogradely labeled GABAergic cells did not colocalize, which is consistent with existing literature (Stamp and Semba, 1995).

These data demonstrate that, in contrast to vGLUT2+ inputs, the majority of the retrogradely labeled neurons (62%) in the purely GABAergic PVT afferent regions are located in the forebrain (BNST, ZI, TRN, and hypothalamus), with only a small proportion situated in the brainstem (Fig. 6*B*,*C*).

### Brain regions with dual, GABAergic, and glutamatergic PVT-projecting neurons

Following viral injections into the PVT of vGAT-Cre and vGLUT2+ mice, we found that 28.43% of the retrogradely labeled vGLUT2+ cells and 30.09% of the vGAT+ cells were confined to brain regions that provided both glutamatergic and GABAergic innervation to the PVT. In the anterior and medial amygdaloid region (Figs. 2*C*,*D*, 5*B1*,*2*; Ant-med amy, 5.57% ± 1.15% glutamatergic, 3.79% ± 0.42% GABAergic), more precisely in the anterior cortical amygdaloid nucleus (ACo), the dorsal and ventral anterior amygdaloid areas (AAD and AAV), the lateral olfactory tract (LOT), and the peri-LOT border region, which showed PV-immunopositivity (Kemppainen and Pitkänen, 2000), as well as the anterodorsal and posterodorsal nuclei of the medial amygdala.

In the hypothalamic preoptic area (POA, 8.85% ± 0.77% glutamatergic, 3.54% ± 1.35% GABAergic), we also found both GABAergic and glutamatergic neurons (Figs. 2*B*, 5*A1*,*2*), although they were present with moderate density in the medial (MPOA) and lateral (LPOA) preoptic regions, forming less dense populations compared with the glutamatergic neurons in the nearby MnPO. Neurons of both types were consistently found in smaller numbers in the anterior hypothalamic area (AHA), the tuber cinereum region, and the medial tuberal nucleus (MTu), as well as in the posterior hypothalamic area (PH).

In the brainstem, we identified two anteroposteriorly organized columns of retrogradely labeled cells: the periaqueductal gray-laterodorsal tegmental nucleus (PAG-LDTg) column and the deep mesencephalic area-pedunculopontine tegmental nucleus column (DpMe-PPTg). Within the PAG-LDTg column, both excitatory (glutamatergic) and inhibitory (GABAergic) neuronal populations were observed, with the periaqueductal gray matter (PAG) emerging as the most significant input region with dual projection (16.13% ± 2.12% glutamatergic, 8.58% ± 1.56% GABAergic; Figs. 2*J*, 5*D1*,*2*). The ventrolateral part of the PAG (vlPAG) contained the highest density of labeled cells for both neuronal types. The laterodorsal tegmental nucleus (LDTg), a cholinergic structure confirmed by choline acetyltransferase (ChAT) immunostaining, also exhibited significant input to the PVT, though ChAT-immunopositive neurons were not retrogradely labeled in this region (Figs. 2*K*, 5*E1*,*2*). Glutamatergic cells were predominantly located ventrally, while GABAergic neurons were found more dorsally in a subventricular position, with minimal overlap between the two populations.

In the DpMe-PPTg column, we observed labeled glutamatergic and GABAergic neurons in the deep mesencephalic area (DpMe), accounting for 6.30% ± 1.72% and 11.43% ± 1.87% of the labeled cells, respectively (Figs. 2*H*,*I*, 5*C1*,*2*). Glutamatergic neurons were primarily located in the anterior, lateral portion of the region (mesencephalic reticular formation), while GABAergic cells were concentrated in the posterior, medial portion. Similar to the LDTg column, there was no complete overlap between the excitatory and inhibitory populations in the DpMe.

Additionally, a small number of retrogradely labeled neurons were sporadically observed in the cuneiform nucleus and the median/paramedian raphe region. It is important to note that, while the labeled cells were grouped according to anatomical boundaries for clarity, these populations formed coherent columns that spanned multiple brainstem regions along an anteroposterior axis.

These data show that the most prominent dual, excitatory, and inhibitory input to PVT arise within and around the periaquaductal region of the brainstem with additional scattered dual projection from the hypothalamus (Fig. 6*A*).

### Control experiments: retrograde injection sites confined to the mediodorsal thalamus and/or the lateral habenula

To eliminate the potential for misidentifying regions with retrogradely labeled cells, which may arise from the involvement of the MD and/or the LHb in individual PVT injections, we conducted a comprehensive analysis of selective MD and/or LHb injections in both vGAT-Cre and vGLUT2-Cre mouse models (Fig. S1). When the injection sites were confined to the MD/LHb (Fig. S1*A,F*), we observed distinct retrograde labeling patterns in the two Cre lines. In vGLUT2-Cre mice (*n* = 3), glutamatergic neurons were labeled in the perifornical area (PeF; Fig. S1*B*) and the interpeduncular nucleus (IPN; Fig. S1*C*).

In vGAT-Cre mice (*n* = 6), a significant number of retrogradely labeled GABAergic neurons were detected in the ventral pallidum (VP; Fig. S1*G*), substantia innominata (SI), GPi (Fig. S1*H*), IPN (Fig. S1*I*), ventral tegmental area (VTA), substantia nigra pars reticulata (SNR; Fig. S1*J*), and in some cases, the suprachiasmatic nucleus (SCh). These regions have been confirmed in the literature to provide innervation to the MD or LHb (Groenewegen, 1988; Kuroda and Price, 1991; Groenewegen et al., 1993; Hong and Hikosaka, 2008).

For selective PVT injections, we did not observe a significant number of labeled neurons in the mentioned areas. Similarly, in control experiments with selective MD/LHb injections (Fig. S1*C,D*), the regions identified as PVT inputs (see above) lacked labeled neurons (Zahm and Root, 2017).

### Parcellation of PVT using calretinin (CR) as a neurochemical marker

**Figure 7.**
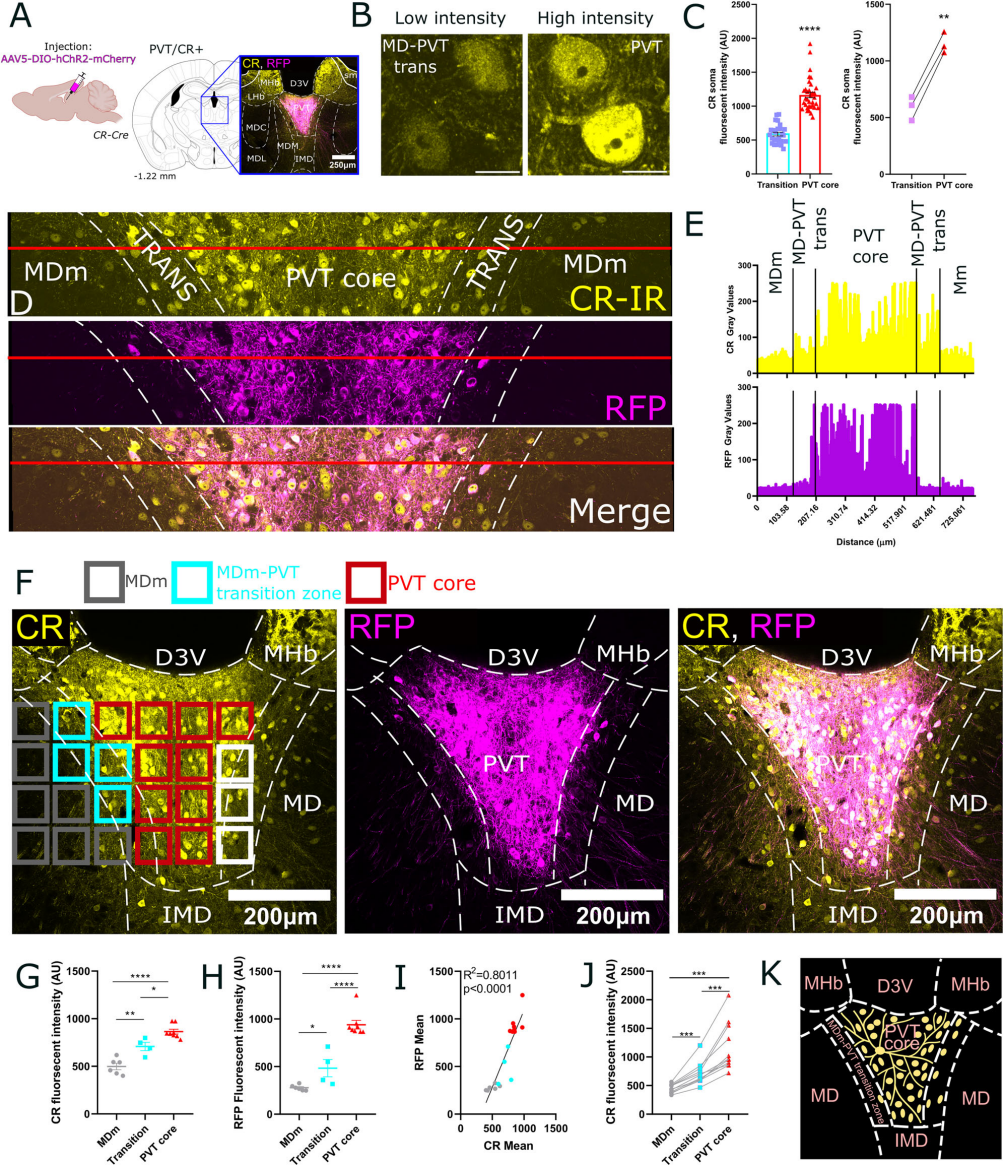
Delineation of PVT based on calretinin immunostaining. ***A***, Schematic representation of the experimental design, including the stereotactic plane and a low-power image of the virus injection site. ***B***, High-power confocal images of calretinin-immunostained cells in the core region of the PVT compared with the transition zone between the MD and PVT. ***C***, Statistical comparison of calretinin immunofluorescence intensity in the PVT core versus the transition zone. ***D***, Confocal images showing calretinin immunostaining (top, yellow), virus injection targeting the core of the PVT in a CR-Cre mouse (middle, magenta), and their overlay (bottom). Note that the virus injection is largely restricted to the core of the PVT, and virus-labeled dendrites do not extend into the transition zone. The red line indicates the region used for CR intensity measurements. ***E***, Fluorescence intensity measurements along the red line shown in ***D***, including calretinin immunostaining (top) and mCherry intensity for virus labeling (bottom). ***F***, Systematic sampling of the PVT core, transition zone, and MD using isotropic voxels. The color-coded squares indicate their assignment: PVT core (red), transition zone (blue), MD (gray). Squares omitted from the analysis are shown in white. ***G***, ***H***, Statistical comparison of CR fluorescence intensity among the three regions (*n* = 21 squares). ***I***, Correlation analysis between the fluorescence intensity of calretinin immunostaining and virus labeling. ***J***, Statistical comparison of CR fluorescence intensity among the three regions in *n* = 11 mice. ***K***, Schematic summary presentation of the distinguished regions. Data are represented as mean ± SEM. *p < 0.05, **p < 0.01, ***p < 0.001, ****p < 0.001. CR, calretinin; CR+, calretinin-positive neuron,; MHb, medial habenula; D3V, third ventricle; MD, mediodorsal thalamus; IMD, intermediodorsal thalamic nucleus; MDm, medial part of the mediodorsal thalamic nucleus; MDm–PVT trans, transition zone between the medial division of the mediodorsal thalamus and the paraventricular thalamus; AAV, adeno-associated virus.

Next, we aimed to examine the regional specificity of the afferents in PVT arising from the brain centers identified by the retrograde tracing experiments. Using immunohistochemistry and in situ hybridization, previous studies identified the PVT as a region containing CR-expressing neurons both in mice (Arai et al., 1994; Mátyás et al., 2018; Gao et al., 2023; Jász et al., 2025) and humans (Cicchetti et al., 1998; Mátyás et al., 2018). To confirm these findings in CR-Cre transgenic mice, we injected AAV5-DIO-mCherry into the PVT (*n* = 3 mice), selectively labeling CR+ neurons with mCherry (Fig. 7*A*; Tables 1, 2). Next we performed double immunofluorescent staining for the virus labeling and CR. Confocal microscopy revealed that 96.53% of mCherry-expressing neurons were CR immunoreactive (501 out of 519 mCherry+ neurons in *n* = 3 mice).

In the CR immunostaining, we observed that the central sectors of the PVT (referred to as the PVT core) contained densely packed CR+ cells whereas the border zone with the MDm (referred as MDm–PVT transition zone; Fig. 7*B*,*C*) contained scattered CR-containing cells (Mátyás et al., 2018). Fluorescent intensity of CR+ cell bodies (Fig. 7*B*,*C*) in the PVT core was significantly higher than that of neurons in the MDm–PVT transition zone (unpaired *t* test, *t*_(59)_ = 10.96, *p* < 0.0001, MDm–PVT transition zone: *n* = 26 cells; PVT core: *n* = 35 cells; *n* = 3 mice, Fig. 7*C*). This effect was consistently observed across all mice (paired *t* test, *t*_(2)_ = 23.31, *p* = 0.0018; Fig. 7*C*). Interestingly, AAV-transduced mCherry expression was largely confined to the PVT core and did not extend to the MDm–PVT transition zone (Fig. 7*D*,*E*). Line intensity profile analysis revealed a striking increase in CR immunofluorescence intensity in the PVT core compared with the MDm–PVT transition zone, highlighting a pronounced difference in CR expression levels between these regions (Fig. 7*D*,*E*). RFP fluorescence values also dropped sharply at the border of the core and the transition zone and were absent in the MDm–PVT transition zone (Fig. 7*D*,*E*). The vast majority of virus-labeled PVT core cells apparently confined their dendrites to the core and did not extend to the transition zone (Fig. 7). Although many dendrites of core PVT neurons appeared to remain within the CR-dense region, we cannot exclude the possibility that some of them extend into adjacent transition zones, as suggested by previous studies (Zhang et al., 2009).

To systematically assess the intensity of CR immunoreactivity in the MD, MD–PVT transition zone, and PVT core, confocal images were acquired, and a 4 × 6 montage was generated at 60× magnification to capture both RFP-expressing and CR immunoreactive neurons in these regions (Fig. 7*F*). These brain areas were sampled along the *x* and *y* axes at 100 μm intervals, with imaging windows (ROI) of 89.51 μm × 89.51 μm and a *z*-step size of 0.09 μm/pixel. Based on the anatomical position of each ROI, we classified them as MDm (gray), MDm–PVT transition zone (cyan), or PVT core (red; Fig. 7*F*). Comparing the CR fluorescent intensity across ROIs classified as MD, MDm–PVT transition zone, and PVT core (Fig. 7*F*), we observed a significant increase in fluorescent intensity from the MDm to the transition zone and further to the PVT core (Fig. 7*F*,*G*; one-way ANOVA, *F*_(2,15)_ = 36.83, *p* < 0.0001; Tukey's post hoc; MDm vs transition zone, *p* = 0.0025; MDm vs PVT, *p* < 0.0001). The PVT core showed significantly increased CR fluorescent intensity compared with the transition zone (Fig. 7*F*,*G*; Tukey's post hoc; transition zone vs PVT, *p* = 0.0146). Comparing RFP fluorescent intensities between MDm, MDm–PVT transition zone, and PVT core regions yielded similar results (Fig. 7*F*,*H*; one-way ANOVA, *F*_(2,15)_ = 54.75, *p* < 0.0001; Tukey's post hoc; MDm vs transition zone, *p* = 0.0454; MDm vs PVT *p* < 0.0001; transition zone vs PVT *p* < 0.0001). In line with this observation, we found a significant positive correlation between CR and RFP fluorescent intensities (Spearman's correlation, *r* = 0.9071, *p* < 0.0001; Fig. 7*I*).

To validate this pattern at the population level across multiple mice and immunohistochemical experiments, we analyzed CR fluorescence (*n* = 11 mice) using a repeated-measures ANOVA (Fig. 7*J*), confirming a significant regional effect (*F*_(1.091,10.91)_ = 40.12, *p* < 0.0001). Post hoc comparisons showed a graded increase in CR intensity from MDm to the transition zone and PVT core (*p* ≤ 0.0005; Fig. 7*J*). These findings demonstrate a consistent regional distribution, with the highest CR fluorescence in the PVT core, followed by the transition zone and MDm.

**Figure 8.**
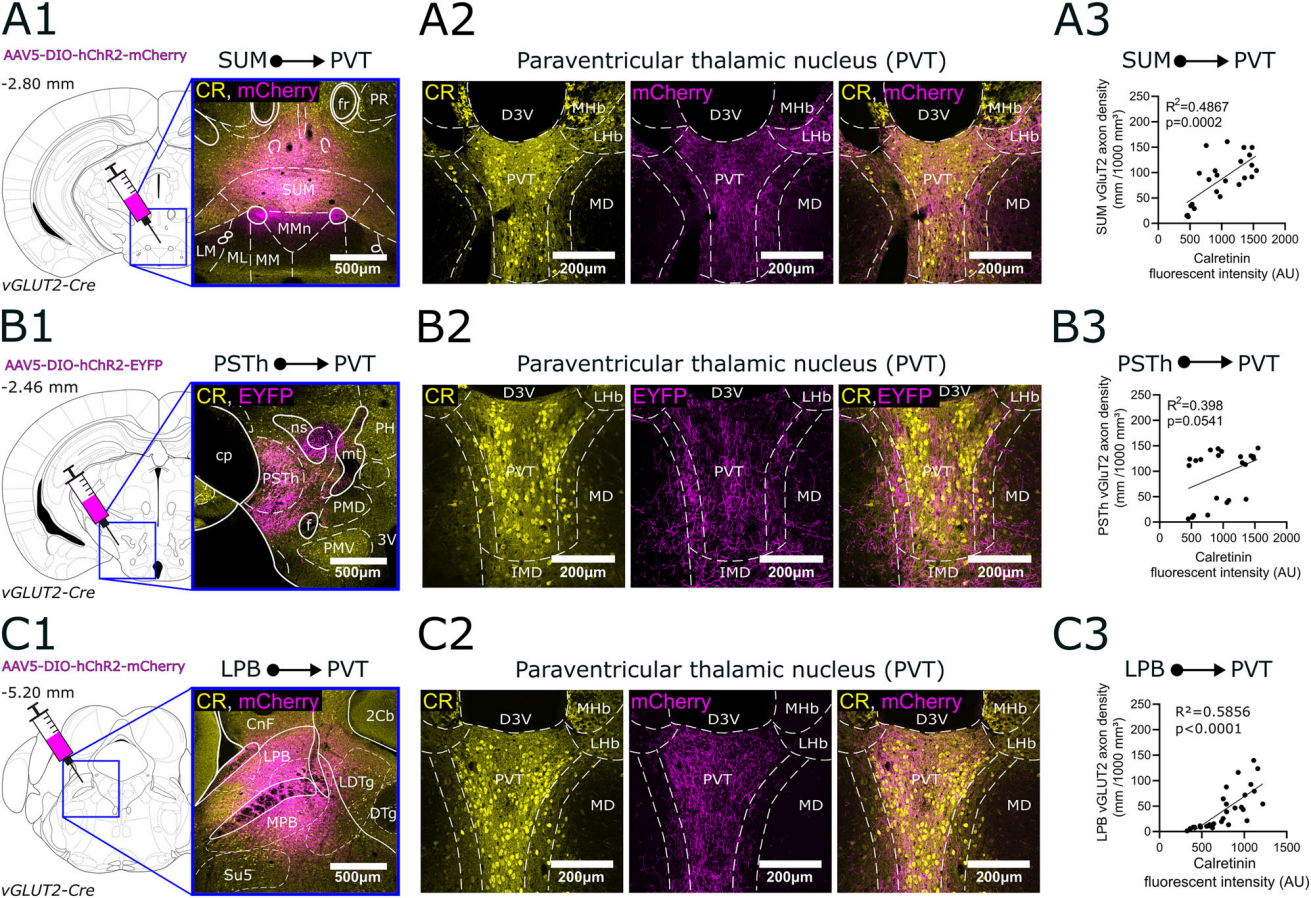
Regional selectivity of subcortical glutamatergic (vGluT2+) inputs within the PVT. ***A1–C1***, The positions of each injection site (MPOA, PSTh, SUM, LPB) are shown in the stereotaxic brain atlas, along with representative confocal images of viral expression in vGLUT2-Cre animals. ***A2***, Regional selectivity of the SUM inputs in the PVT. Left, CR immunostaining; middle, SUM fibers with mCherry, right column overlay. ***A3***, Correlations between axon density and the fluorescence intensity of CR immunostaining. AU, arbitrary units. ***B2***,***3***, Same for PSTh axons. While PSTh afferents are selective for PVT they largely avoid CR+ somata, thus there is no significant correlation between axon density and fluorescent intensity. ***C2***,***3***, Same for LBP axons. LBP axons are highly selective for the core region of PVT and their density display significant correlation with CR fluorescent intensity. 2Cb, 2nd cerebellar lobe; 3V, 3rd ventricle; AAV5, adeno-associated virus serotype 5; aca, anterior commissure anterior part; acp, anterior commissure posterior part; ADP, anterodorsal preoptic nucleus; CnF, cuneiform nucleus; cp, cerebral peduncle; CR, calretinin; D3V, dorsal part of the 3rd ventricle; DIO, double-floxed inverted open reading frame; DTg, dorsal tegmental nucleus; EYFP, enhanced yellow fluorescent protein; fr, fasciculus retroflexus; hChR2, human channelrhodopsin 2; MD, mediodorsal nucleus; MHb, medial habenula; MM, medial mammillary nucleus medial part; MMn, medial mammillary nucleus medial part; ML, medial mammillary nucleus lateral part; MPA, medial preoptic area; MPB, medial parabrachial nucleus; MPOA, medial preoptic area; mt, mammillothalamic tract; LDTg, laterodorsal tegmental nucleus; LHb, lateral habenula; LPB, lateral parabrachial nucleus; LM, lateral mammillary nucleus; LPO, lateral preoptic area; ns, nigrostriatal bundle; PH, posterior hypothalamic area; PMD, dorsal part of the premammillary nucleus; PMV, ventral part of the premammillary nucleus; PR, prerubral area; PSTh, parasubthalamic nucleus; Su5, supratrigeminal nucleus; SUM, supramammillary nucleus; vGLUT2, vesicular glutamate transporter type.

**Figure 9.**
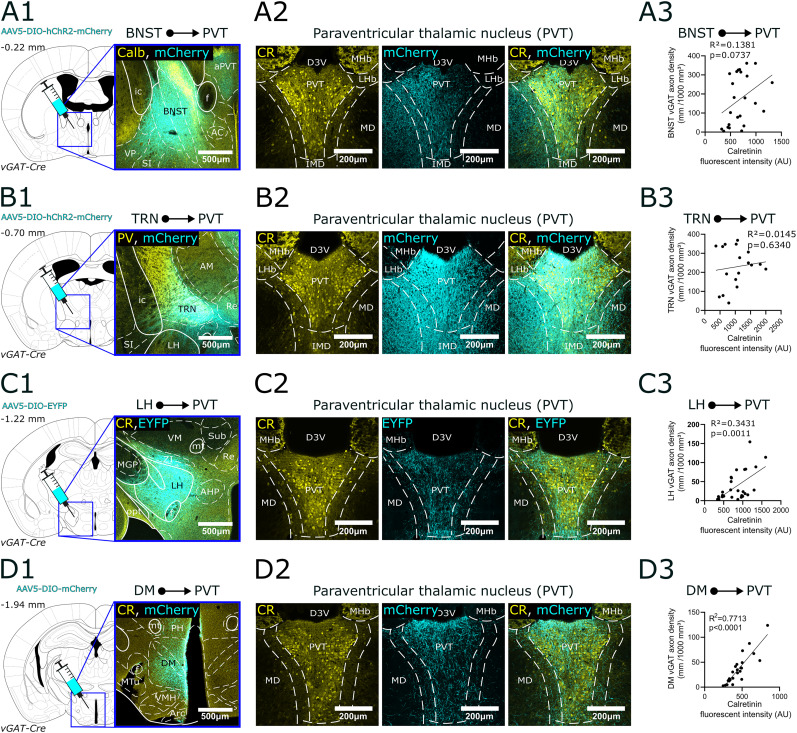
Regional selectivity of subcortical GABAergic (vGAT+) inputs within the PVT. ***A1–D1***, The position of each injection site (BNST, TRN, LH, DM) are shown in the stereotaxic brain atlas, along with representative confocal images of viral expression in vGAT-Cre animals. ***A2–B3***, Regional selectivity of the BNST and TRN inputs in the PVT. Left, CR immunostaining (yellow); middle, anterogradely labeled fibers, mCherry or EYFP (cyan); right, overlay; far right, correlations between axon density and the fluorescence intensity of CR immunostaining. AU, arbitrary units. BNST and TRN fibers do not display regional selectivity in PVT. ***C1–D3***, Same for LH and DM inputs. Both hypothalamic inputs are highly selective for the core region of the PVT. AAV5, adeno-associated virus serotype 5; AC, anterior commissure nucleus; AM, anteromedial thalamic nucleus; AHP, anterior hypothalamic nucleus posterior part; aPVT, anterior PVT; Arc, arcuate nucleus; BNST, bed nucleus of the stria terminalis; Calb, calbindin; CR, calretinin; D3V, dorsal part of the 3rd ventricle; DIO, double-floxed inverted open reading frame; DM, dorsomedial hypothalamic nucleus; EYFP, enhanced yellow fluorescent protein; f, fornix; ic, internal capsule; hChR2, human channelrhodopsin 2; IMD, intermediodorsal thalamic nucleus; LH, lateral hypothalamus; LHb, lateral habenula; MD, mediodorsal thalamic nucleus; MGP, medial globus pallidus; MHb, medial habenula; mt, mammillothalamic tract; MTu, medial tuberal nucleus; opt, optic tract; PH, posterior hypothalamic area; PV, parvalbumin; Re, reuniens nucleus; SI, substantia innominata; Sub, submedius thalamic nucleus; TRN, reticular thalamic nucleus; vGAT, vesicular GABA transporter; VM, ventromedial thalamic nucleus; VMH, ventromedial hypothalamic nucleus; VP, ventral pallidum.

**Figure 10.**
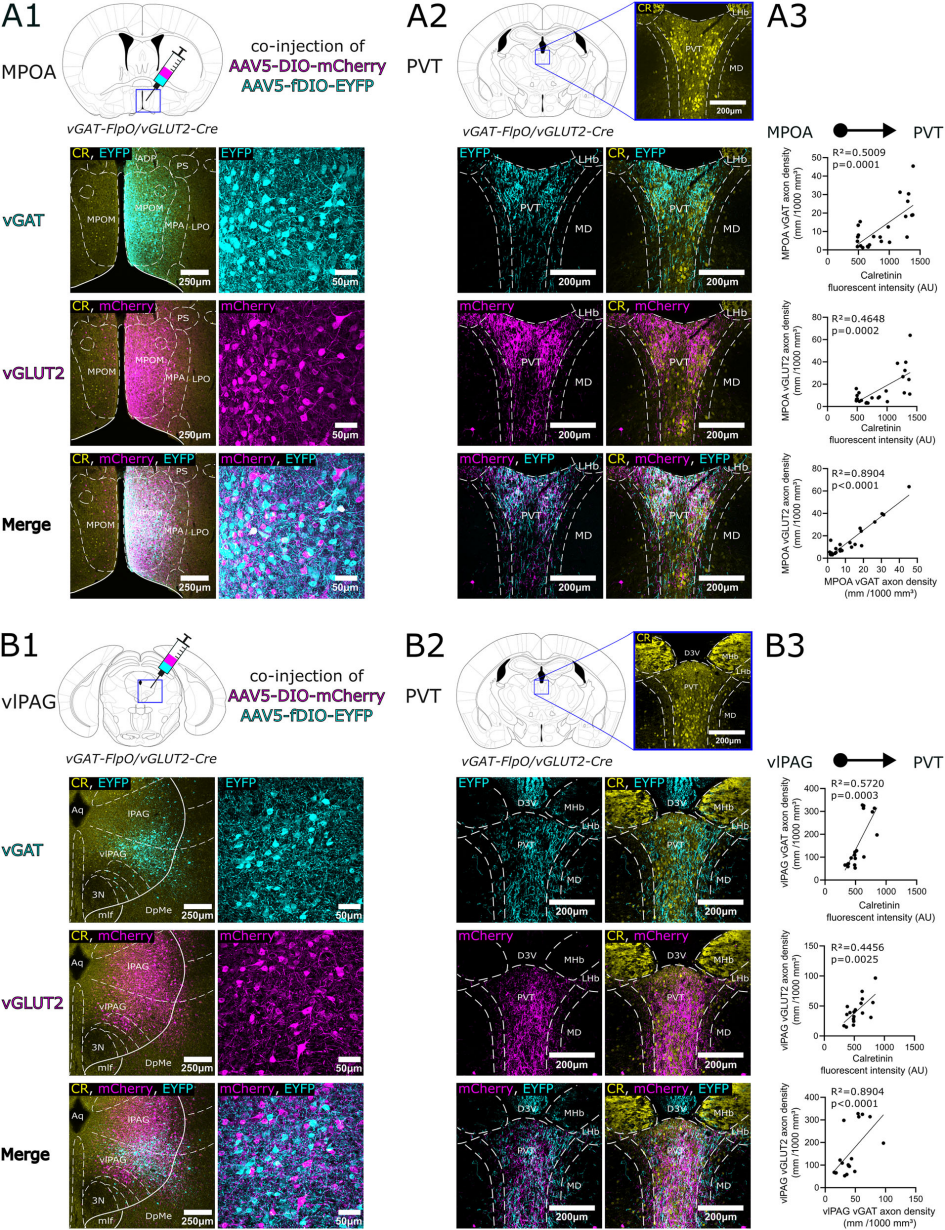
Convergence and selectivity of mixed GABAergic and glutamatergic inputs within the PVT. ***A1***, Top, A schematic diagram of the experimental setup. A mixture of FlpO and Cre-dependent viruses were injected into the MPOA of a double transgenic vGAT-FlpO/vGLUT2-Cre animal. Bottom, The expression of the two viruses at the injection site with 10× and 20× magnification; the composite image shows that there is no colocalization between the reporter proteins labeling GABAergic and glutamatergic cells. ***A2***, Top, The position of the PVT section in the stereotaxic brain atlas. Bottom, first row, AAV5-fDIO labeled vGAT+ fibers (left) of MPOA origin in the PVT and overlay of the fibers with CR immunostaining; second row, AAV5-DIO labeled vGLUT2+ fibers in the same animal; third row, overlay. ***A3***, From top to bottom, Correlation between GABAergic MPOA axon density and CR fluorescence intensity. Correlation between glutamatergic MPOA axon density and CR fluorescence intensity. Correlation between the GABAergic MPOA axon density and the glutamatergic MPOA axon density in the PVT. ***B1–B3***, The same for the mixed GABAergic, glutamatergic inputs from vlPAG. The two components of both inputs converge and show high selectivity for the core region of PVT. 3N, oculomotor nucleus; AAV5, adeno-associated virus; AAVDJ, adeno-associated virus serotype DJ; AU, arbitrary units; Aq, cerebral aqueduct; CR, calretinin; D3V, dorsal part of the 3rd ventricle; DIO, double-floxed inverted open reading frame; DpMe, deep mesencephalic area; EYFP, enhanced yellow fluorescent protein; MD, mediodorsal thalamic nucleus; MHb, medial habenula; mlf, medial longitudinal fasciculus; LHb, lateral habenula; lPAG, lateral periaqueductal gray; PVT, paraventricular thalamic nucleus; vGAT, vesicular GABA transporter; vGLUT2, vesicular glutamate transporter 2; vlPAG, ventrolateral periaqueductal gray.

**Figure 11.**
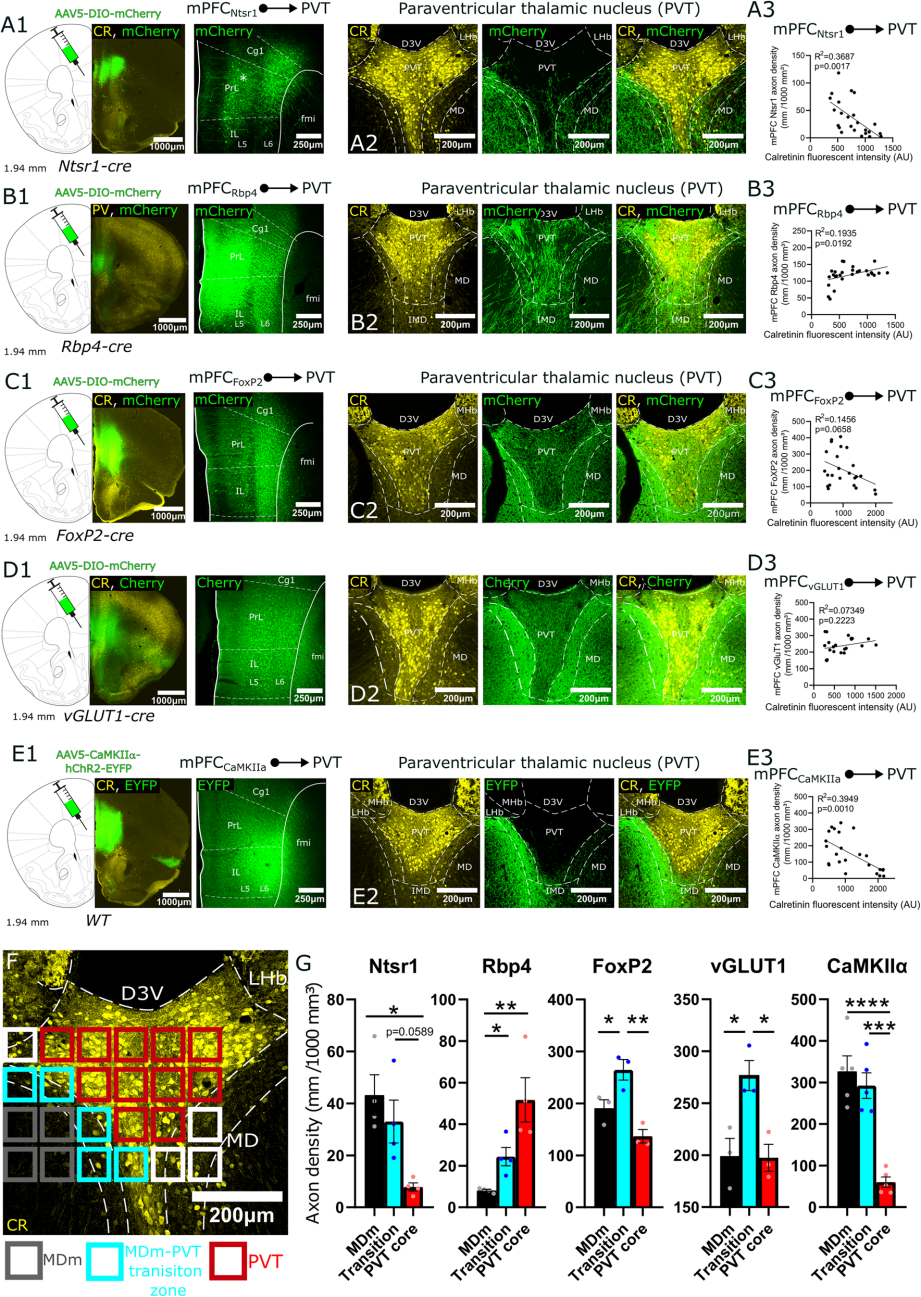
Regional selectivity of cortical inputs from different layers of the mPFC in the PVT. ***A1***, The position of the mPFC in the stereotaxic brain atlas is shown, along with representative confocal images from the injection site at 4× and 10× magnification in an L6 specific Ntsr1-Cre animal. Note that in the mPFC of Ntsr1-Cre animals L5 cells are also labeled (asterisk) but these cells are known to project to subcortical structures other than the thalamus (Fig. S7; Babiczky and Matyas, 2022). ***A2***, Regional selectivity of Ntsr1 inputs in the PVT together with CR immunostaining. Ntsr1 fibers avoid the CR+ core region of the PVT but heavily innervate the transition zone and the MD. ***A3***, Significant negative correlations between Ntsr1 axon density and the fluorescence intensity of CR immunostaining. AU, arbitrary units. ***B1–B3***, The same for layer 5 selective Rbp4 animals. Rbp4 input is complementary to Ntsr1 afferentation, and its density positively correlates with CR fluorescence intensity. ***C1–C3***, The same for L5/L6 selective FoxP2 mice. Both the transient zone and the core region are innervated with more fibers. ***D1–D3***, The same in vGluT1-Cre mice. Dense vGluT1+ fibers are concentrated in the transition zone, with lower but comparable innervation in the MDm and PVT core. ***E1–E3***, The same in a wild animal after the injection of AAV5-CaMKIIα promoter viral construct. PVT core afferents are conspicuously absent. ***F***, Representative images showing the position of the squares used for quantitative measurements of cortical axonal densities and CR+ fluorescence intensities. Color coding as in Figure 7*F*. ***G***, Quantification of axon density across the MDm, MDm–PVT transition zone, and PVT core in Ntsr1-Cre, Rbp4-Cre, FoxP2-Cre, vGLUT1-Cre, and CaMKIIα-driven viral labeling. AU, arbitrary units; AAV5, adeno-associated virus serotype 5; CaMKIIα, calcium/calmodulin-dependent kinase IIα; Cg1, cingulate cortex; CR, calretinin; D3V, dorsal part of the 3rd ventricle; DIO, double-floxed inverted open reading frame; EYFP, enhanced yellow fluorescent protein; fmi, forceps minor; FoxP2, Forkhead box protein P2; hChR2, human channelrhodopsin 2; IMD, intermediodorsal thalamic nucleus; IL, infralimbic cortex; MD, mediodorsal thalamic nucleus; MHb, medial habenula; Ntsr1, neurotensin receptor 1; Rbp4, retinol-binding protein 4; LHb, lateral habenula; PrL, prelimbic cortex; WT, wild type.

**Figure 12.**
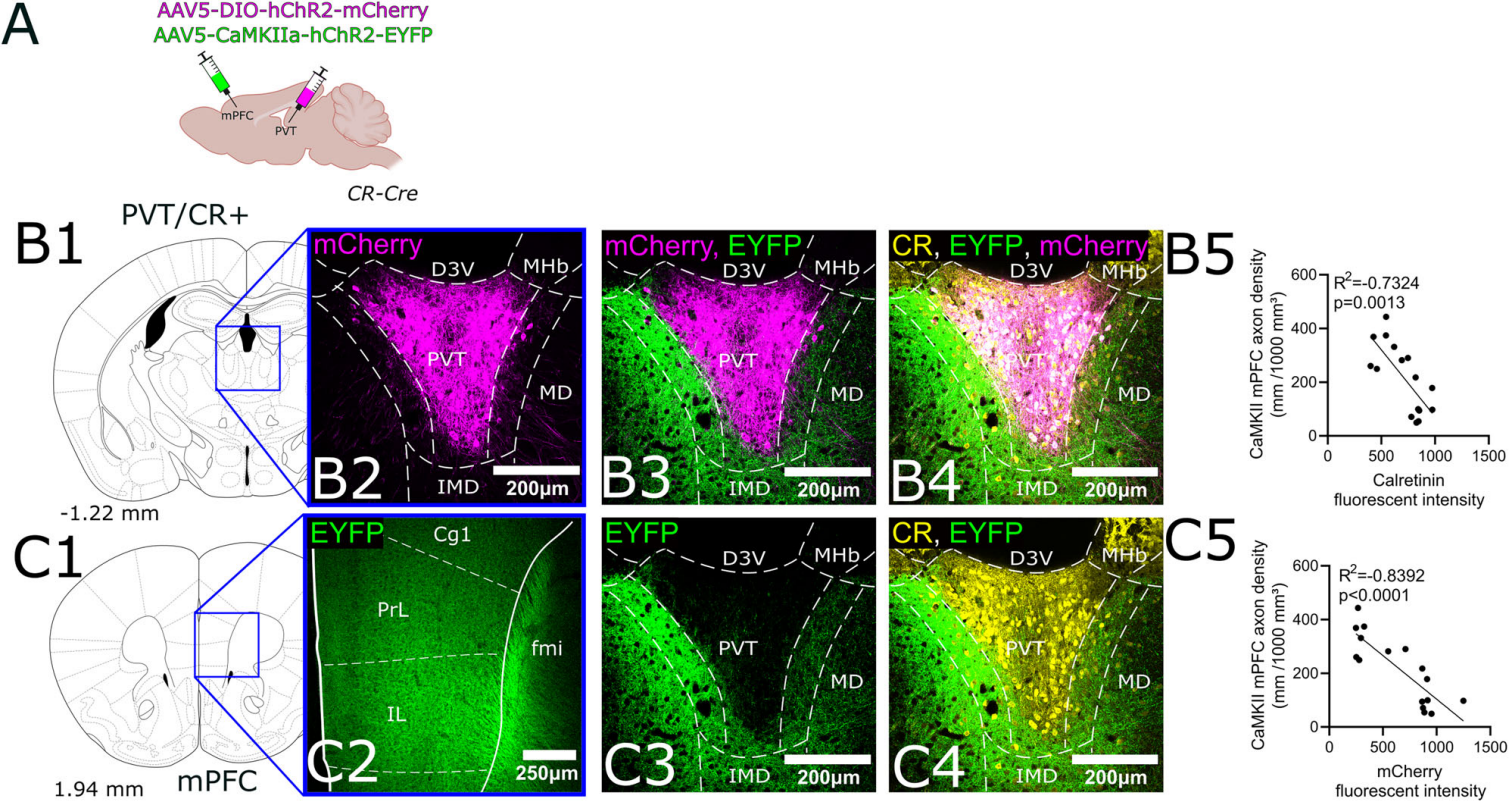
Segregation of cortical inputs labeled by CaMKIIα and the core region of the PVT labeled by viral tracing. ***A***, Schematic representation of the experimental design. AAV5-DIO-mCherry was injected into the PVT of CR-Cre mice to selectively label CR+ core neurons, while AAV5-CaMKIIα-hChR2-EYFP was injected into the mPFC to trace cortical projections. ***B1***,**2**, Schematic coronal section of the PVT (***B1***) and a confocal image of the mCherry injection site in the PVT (***B2***), showing selective labeling of CR+ core neurons. ***B3***, Overlay of mCherry-labeled CR+ neurons and EYFP-expressing cortical projections labeled by the AAV5-CaMKIIα viral construct. ***B4***, Triple overlay of CR immunostaining, EYFP-labeled cortical projections, and mCherry-labeled CR+ neurons. Note complementary labeling patterns between CaMKIIα-labeled cortical axons and CR+ core neurons in the PVT. ***B5***, Quantification of axonal density in relation to CR fluorescence intensity in the PVT, showing a negative correlation, indicating that cortical CaMKIIα+ projections avoid the PVT core, where CR+ neurons are concentrated. ***C1*–*3***, Cortical projections labeled by the CaMKIIα promoter in a CR-Cre animal. Schematic coronal section of the mPFC (***C1***) and a confocal image of the EYFP injection site in the mPFC (***C2***), showing viral expression in the PrL and IL regions. ***C3***, EYFP-labeled cortical projections in the PVT and MDm, showing preferential innervation of the MDm and the MDm–PVT transition zone. ***C4***, Overlay of EYFP-labeled cortical projections and mCherry-labeled CR+ neurons, demonstrating the segregation between cortical axons and the CR+ core region of the PVT. ***C5***, Quantification of axonal density in relation to mCherry-labeled CR+ neuron intensity, revealing a negative correlation, further supporting the finding that mPFC CaMKIIα+ projections primarily target the MDm and MDm–PVT transition zone while avoiding the PVT core. Scale bars: 200–250 µm. These findings reveal that cortical projections labeled with the CaMKIIα promoter preferentially target the MDm and the MDm–PVT transition zone, while avoiding the PVT core, which is densely populated by CR+ neurons. PVT, paraventricular thalamus; CR, calretinin; mPFC, medial prefrontal cortex; PrL, prelimbic cortex; IL, infralimbic cortex; MDm, medial subdivision of the mediodorsal thalamus; EYFP, enhanced yellow fluorescent protein; mCherry, red fluorescent protein variant; D3V, dorsal third ventricle; IMD, intermediodorsal nucleus; MHb, medial habenula.

In summary, quantitative assessment of CR-immunofluorescence staining revealed a dense inner or “core” region within the PVT, characterized by neurons exhibiting high levels of CR intensity and a transition zone between the PVT and MD that displayed lower fluorescent signals (Fig. 7*K*). We used this parcellation method to delineate the regional selectivity of various subcortical and cortical inputs to the PVT (Figs. 8–12). For quantitative evaluations of the inputs, we calculated the total length of the axons in a given *z*-stack and determined the axon density as the ratio of total axon length to the volume of the *z*-stack (see Materials and Methods).

### Regional selectivity of the main vGLUT2+ afferents in PVT

In order to examine the exact position of the afferent fibers relative to the PVT/CR+ population, we conducted anterograde tracing from the brain centers identified by retrograde tracing. This also served as a validation of our retrograde findings. We injected anterograde Cre-dependent adeno-associated virus (AAV) vectors in vGLUT2-Cre mice to the SUM (*n* = 4), PSTh (*n* = 4), and LPB (*n* = 9; Fig. 8; Tables 1, 2).

For SUM injections, three out of four experiments were successful, while one injection did not result in viral expression. The successful injections remained within the SUM region (Fig. 8*A*1), with minimal spillover into the dentate gyrus, PAG, and mammillary body. Dense axonal innervation was observed in the PVT core (Fig. 8*A2*,*3*), while the MDm–PVT transition zone exhibited lower axonal density, and projections to the MDm were sparse (Fig. S2*A*). Axon density of SUM inputs showed a significant positive correlation with CR fluorescence intensity (Fig. 8*A3*).

For PSTh injections, three out of four experiments were successful (Fig. 8*B1*). The excluded injection missed the PSTh in a caudal direction and was therefore not included in the analysis. The successful injections minimally affected the LH, and in two cases, the capillaries along the injection path marked thalamic nuclei above the PSTh. According to the literature, these thalamic nuclei do not send direct innervation to the PVT, which was confirmed by our control experiments (*n* = 2). Inputs from the PSTh innervated the core area of the PVT (Fig. 8*B2*, Fig. S2*B*), but axonal density from the PSTh was also elevated in the MD–PVT transition zone (Fig. 8*B2*,*3*; Fig. S2*B*). PSTh fibers avoided the MD (Fig. 8*B2*, Fig. S2*B*). Since fiber density was also high in the transition zone that display low CR intensity, no significant correlation was found between CR fluorescence intensity and axonal density (Fig. 8*B3*).

In the LPB experiments, seven out of nine injections were successful (Fig. 8*C1*). Unsuccessful injections affected the LPB but also spread extensively to the superior colliculus (SC), PAG, and cuneiform nucleus, so these animals were excluded from the analysis. The successful injections showed uniform results, establishing that the LPB provides robust subcortical glutamatergic input to the PVT (Fig. 8*C2*). Axonal density was strongest in the core of the PVT (Fig. 8*C2*, Fig. S2*C*). Only weak innervation was observed in the MD–PVT transition zone and the MD did not receive input from the LPB (Fig. 8*C2*, Fig. S2*C*). Confocal analysis results showed a robust, significant correlation between axonal density from the LPB and CR fluorescence intensity (Fig. 8*C3*), highlighting the selectivity of LPB inputs to the PVT. Among all examined glutamatergic input regions, the LPB showed the highest axonal density in the PVT, in alignment by the quantitative analysis of retrograde experiments.

Axons from the SUM, PSTh, and LPB were present throughout the entire rostrocaudal extent of the PVT consistent with previous tracing studies in rat and mouse (Goto and Swanson, 2004; Li and Kirouac, 2012). They formed dense, clustered arborizations in the CR+ core region of the PVT. In case of the PSTh, the MD–PVT transition zone also received dense innervation, while axons were extremely rare in the MD region.

### Regional selectivity of the main vGAT+ afferents in PVT

We conducted anterograde viral tracing in vGAT-Cre mice by injecting Cre-dependent AAVs into the BNST (*n* = 4), TRN (*n* = 7), LH (*n* = 10), and DM (*n* = 9; Fig. 9; Tables 1, 2).

For the BNST, three out of four injections were successful. The unsuccessful case was excluded from the analysis because the virus spread to the ventral pallidum (VP) and anterior hypothalamic nuclei. In the successful injections, the AAV was targeted to the posterolateral, posteromedial, and posterointermediate nuclei of the BNST, avoiding the anterior nuclei (Fig. 9*A1*). Parvalbumin immunostaining confirmed that viral expression did not cross into the midline of the TRN (data not shown), confirming the specificity of the injections to the BNST. BNST fibers were distributed the core region, the transition zone as well is in MDm (Fig. 9*A2*, Fig. S3*A*). As a result no significant positive correlation was found between CR fluorescence intensity and axon density from the BNST (Fig. 9*A3*).

For the TRN, our retrograde experiments showed that GABAergic TRN neurons projecting to the PVT are located in the anterior ventromedial sector (Fig. 9*B1*). Accordingly, our viral injections targeted this area. Out of seven injections, three were successful, with injection sites remaining within the anatomical boundaries of the TRN, as confirmed by PV immunostaining (Fig. 9*B1*). The virus transfected the entire anteromedial TRN sector, as shown in Figure 9*B1*. According to literature (Cornwall and Phillipson, 1988a; Chen and Su, 1990; Pinault and Deschênes, 1998), TRN neurons projecting to the MD are located next to PVT-projecting cells medially (Hádinger et al., 2023). Our injection resulted in the transfection of both populations in these experiments. Accordingly, axons from the TRN densely and evenly innervated the MD, the MD–PVT transition zone, and PVT core (Fig. 9*B2*, Fig. S3*B*). No significant correlation was found between CR fluorescence intensity and the TRN axon density projecting to the PVT (Fig. 9*B3*). Control injections (*n* = 2) targeting the motor or sensory sectors of the TRN produced no PVT projection, further supporting the specificity of this connectivity and aligning with previous findings in the literature (Pinault and Deschênes, 1998; Halassa et al., 2014; Hádinger et al., 2023).

In the hypothalamus, the LH was the region with the largest number of input neurons in vGAT-Cre animals. Out of the 10 LH injections, six were successful (Fig. 9*C1*), while four cases did not remain within the anatomical boundaries of the LH or missed the target. Among the six successful injections, the three largest covered the entire anteroposterior axis of the LH. The larger injections might have slightly affected neighboring areas like the ZI but more selective injections limited strictly to the LH (*n* = 2) showed a similar axonal pattern. The vast majority of LH terminals were restricted to the core region with minimal spread to the transition zone (Fig. 9*C2*, Fig. S3*C*). The MD did not contain noticeable amount of fibers. This resulted in a significant positive correlation between CR fluorescence intensity and axon density of LH inputs Figure 9*C3*.

For the DM, five out of nine injections were successful (Fig. 9*D1*), while in other cases, the virus spread to surrounding areas such as the ZI, SUM, PSTh, and LH. In all five successful injections, a dense axonal network was observed in the PVT (Fig. 9*D2*). Similar to LH fibers axonal projections from DM were primarily concentrated in the core of the PVT, particularly in the periventricular region (Fig. 9*D2*, Fig. S3*D*). DM formed even less dense axonal network in the MD–PVT transition zone compared with the LH inputs (Fig. 9*D2*). No axonal labeling was observed in the MD (Fig. 9*D2*). In the case of the DM, a robust, significant correlation was found between CR fluorescence intensity and axon density (Fig. 9*D3*), confirming the selective innervation of the PVT from the DM.

These data show that both GABAergic HT inputs (LH and DM) display high selectivity for the core region of PVT; the BNST input, however, targets both the MDm and the PVT. Inability to selectively label the PVT-projecting sector of TRN likely explains the lack of specificity in this vGAT input.

### Convergence of vGLUT2+ and vGAT+ inputs originating from the same subcortical centers in the PVT core

In our retrograde tracing experiments, we identified several brain regions that contain both GABAergic and glutamatergic neurons projecting to the PVT (Figs. 5, 6). From the hypothalamus, the MPOA, and from the brainstem, the vlPAG emerged as prominent candidates for further investigation due to the high number of retrogradely labeled neurons found in these areas. Next we aimed to examine the regional selectivity of these two fiber components in the same animals (Fig. 10). To achieve this, we used double transgenic vGLUT2-Cre/vGAT-FlpO mice and injected FlpO- and Cre-dependent AAV5-fDIO-EYFP and AAV5-DIO-hChR2-mCherry vectors into the MPOA (*n* = 3) or vlPAG (*n* = 3; Fig. 10*A1*,*B1*; Tables 1, 2). This approach allowed for the selective expression of EYFP in GABAergic neurons and mCherry in glutamatergic neurons (Fig. 10*A1*,*B1*). Confocal microscopy confirmed that the vGLUT2+ glutamatergic and EYFP+ GABAergic neurons formed distinct populations in the MPOA and PAG (Fig. 10*A1*,*B1*). There was no colocalization between the reporter proteins marking these neuronal populations in the PAG. In the MPOA, however, we observed that a few neurons (<10%) expressed both EYFP and RFP (Fig. 10*A1*,*B1*).

Following MPOA injections, both vGAT+ and vGLUT2+ inputs selectively targeted the PVT core (Fig. 10*A2*, Fig. S4*A*) with minimal spread to the transition zone. No fibers were observed in MD. In a few cases we observed EYFP and RFP colocalization in the axon terminals, indicating that a subpopulation of MPOA neurons expresses both vGLUT2+ and VGAT+. The axonal densities of both vGLUT2+ and VGAT+ inputs from the MPOA significantly correlated with CR fluorescence intensity, underscoring the selectivity of these inputs (Fig. 10*A3*). The vGAT+ and vGLUT2+ inputs also displayed a positive correlation with each other (Fig. 10*A3*).

Similar to the MPOA, both the vGAT+ and vGLUT2+ components of the vlPAG projection selectively targeted the PVT core, with minimal spread to the transition zone and no fibers in the MDm (Fig. 10*B2*, Fig. S4*B*). Both vGAT+ and vGLUT2+ axonal densities positively correlated with CR fluorescence intensity, as well as with each other (Fig. 10*B3*). Control injections restricted to the dorsolateral PAG and SC resulted in the lack of fibers in PVT.

These data show that the excitatory and inhibitory components of MPOA and vlPAG inputs converge selectively in the CR+ core region of the PVT.

### Layer-specific connectivity between mPFC and the different sectors of PVT

The connections between the medial prefrontal cortex (mPFC) and the PVT play an important role in various behavioral processes, including fear learning, reward-related behaviors, and social behavior (Kirouac, 2015; Penzo and Gao, 2021; Vertes et al., 2022). Thus, next we aimed to uncover the regional selectivity and the layer specificity of the mPFC-PVT and mPFC-MD pathways, according to the CR-based parcellation of the PVT and MD (Fig. 7). To this end, we used different transgenic mouse strains and specific viral constructs. In the Ntsr1-Cre (neurotensin receptor 1, *n* = 6), Rbp4-Cre (retinol-binding protein 4, *n* = 8), FoxP2-Cre (forkhead box protein 2, *n* = 3), and vGLUT1-Cre (*n* = 3) transgenic mouse strains, we injected AAV5-hSyn-DIO-mCherry virus to explore the projection of different cortical layers to the PVT (Fig. 11). We also injected the widely used AAV5-CaMKIIα-hChR2(H134R)-EYFP virus construct that contains a CaMKIIα promoter to express EYFP in wild-type mice (*n* = 5; Otis et al., 2019; Aquino-Miranda et al., 2024; Ma et al., 2024).

In the Ntsr1-Cre transgenic mice, we achieved successful AAV mediated transfection in the mPFC (Fig. 11*A1*). In two cases, fewer than 100 cells were infected, and no axons were detected in the thalamus. The Ntsr1-Cre transgenic mouse strain has been previously utilized successfully for viral labeling of L6 corticothalamic cells in the sensory and motor cortices (DeNardo et al., 2015; Tasic et al., 2016; Hoerder-Suabedissen et al., 2018). In our case, labeled cells were also predominantly located in L6 of the mPFC. Scattered cells were also present more superficially (Fig. S5). Earlier these cells were shown to project to subcortical centers other than thalamus such as the NAc (Babiczky and Matyas, 2022).

Following virus injection in the Ntsr1-Cre mice, the PVT core was almost entirely devoid of labeled fibers, with only a few axons observed in the most medial part of the PVT (Fig. 11*A2*). Innervation was primarily concentrated in the MD–PVT transition zone and the MDm (Fig. 11*A2*). A significant negative correlation was observed between CR fluorescence intensity and Ntsr1+ axon density (Fig. 11*A3*). To confirm the L6 phenotype of Ntsr1+ fibers, we examined the structure of the afferent terminals at high resolution. Consistent with the literature, Ntsr1+ axon terminals in the transition zone and MD exhibited drumstick-like morphology (Fig. S6*A*) confirming their L6 origin (Bourassa and Deschênes, 1995; Hoerder-Suabedissen et al., 2018). In the sensory and motor cortices, Ntsr1+ neurons primarily label L6 corticothalamic neurons, whose axons exclusively innervate the thalamus. Consistent with this, we did not find labeled Ntsr1+ fibers in the brainstem or hypothalamus (Fig. S7*A*).

The Rbp4-Cre transgenic mouse strain is typically used to label L5 cortical neurons (Hoerder-Suabedissen et al., 2018; Babiczky and Matyas, 2022; Hádinger et al., 2023). In the Rbp4-Cre transgenic mouse strain, four injections selectively targeted the mPFC, with their extent limited to the PrL and IL regions (Fig. 11*B1*). In four additional cases, the injection was not mPFC selective and mainly affected the medial and ventral orbitofrontal cortices (MO and VO), as well as the dorsal tenia tectum region. In line with the findings from other cortical regions, we observed in Rbp4-Cre mice that large pyramidal neurons with prominent apical dendrites were labeled in L5 of mPFC (Fig. S5*B*).

The injections in Rbp4-Cre animals resulted in smaller axonal clouds in the PVT compared with subcortical experiments (Fig. 11*B2*). Scant axonal innervation was observed in the MD, and there was a gradual increase in axonal density starting from the MD–PVT transition zone to the PVT core, where axons entered and arborized (Fig. 11*B2*). Among the layer-specific cortical injections, the Rbp4-Cre transgenic mouse strain specific to L5 was the only one where we found a significant positive correlation between CR fluorescence intensity and mPFC axon density in the PVT (Fig. 11*B3*). In order to confirm the L5 phenotype of Rbp4+ fibers, we examined the structure of the afferent terminals at high power. Rbp4+ axon terminals in the PVT core appeared as globular swellings along the axon shaft, consistent with “en passant” boutons, rather than forming “drumstick-like” terminal boutons at axon endings that is a characteristic of the modulatory L6 synaptic transmission (Fig. S6*B*; Ojima, 1994; Hoerder-Suabedissen et al., 2018; Harris et al., 2019). Consistent with the L5 phenotype, we observed Rbp4+ dense axonal labeling in subcortical structures including NAc, VTA, hypothalamus, and brainstem (Deschênes et al., 1994; Guillery and Sherman, 2011; Babiczky and Matyas, 2022).

In the FoxP2-Cre transgenic mouse strain, reporter protein expression was observed in the PrL and IL regions in all three injections (Fig. 11*C1*), mainly in the deeper parts of L5 and in L6, consistent with previous findings (Fig. S5*C*; Babiczky and Matyas, 2022). Both the MDm and MD–PVT transition zone showed dense innervation (Fig. 11*C2*). Compared with them, the PVT core exhibited less dense fibers (Fig. 11*C2*). Unlike the previous two cortical injections, FoxP2+ cortical inputs exhibited denser, more homogeneous distribution, with a topography and morphology consistent with L6 corticothalamic terminals (Fig. 11*C2*). No significant correlation was observed between CR fluorescence intensity and the density of FoxP2+ axons (Fig. 11*C3*). Among all the layer-specific cortical injections, the mPFC-FoxP2 injections resulted in the highest axonal density in the PVT (Fig. 11*C3*). Confocal microscopy revealed that drumstick-like boutons were abundant in the MD and MD–PVT transition zone, while en passant boutons were present in the PVT core.

In the vGluT1-Cre transgenic mouse line (*n* = 3), we injected AAV5-hSyn-DIO-mCherry into the prelimbic (PrL) and infralimbic (IL) regions of the mPFC to label the projections of cortical glutamatergic neurons to the PVT and surrounding thalamic nuclei (Fig. 11*D1*). Robust transfection was observed across all cortical layers (Fig. S5*D*), consistent with the broad expression pattern of vGluT1 in excitatory neurons (Fremeau et al., 2001; Vigneault et al., 2015). Dense axonal labeling was observed in the MDm and in the MD–PVT transition zone, while the PVT core also exhibited strong arborization, although with lower density compared with the transition zone (Fig. 11*E2*). Quantitative analysis of axonal distribution revealed no significant correlation between CR fluorescence intensity and PVT axon density (Fig. 11*D3*). These findings confirm that mPFC-derived glutamatergic projections target both the core and the transition zone of the PVT, with a higher density in the latter, and support an innervation of both the core and transition zones of the PVT arising from broadly distributed cortical excitatory neurons.

Previous reports focusing on the mPFC-PVT pathway used AVVs with CaMKIIα promoter to transduce cortical neurons independently of layers (Otis et al., 2019; Yamamuro et al., 2020; Aquino-Miranda et al., 2024; Ma et al., 2024), since CaMKIIα considered a pan-neuronal marker for cortical projection neurons (Liu and Jones, 1996). To replicate these experiments, we injected AAV5-CaMKIIα-hChR2(H134R)-EYFP virus into the mPFC of wild-type mice. The injections successfully labeled the infralimbic (IL) and prelimbic (PrL) regions (Fig. 11*E1*), where the infected cells were observed in all layers (Fig. S5*E*). Dense axonal network was observed in the MD–PVT transition zone. Interestingly the core region contained only scattered fibers in stark contrast to the Rbp4-Cre injections (Fig. 11*E2*, Fig. S6*E*). Similarly, dense axonal innervation was found in the medial part of the MD (Fig. 11*E2*). A negative correlation was observed between CR fluorescence intensity and the density of mPFC axons labeled with the CaMKIIα promoter virus (Fig. 11*E3*). Presumably due to retrograde transport, we occasionally observed EYFP transfected neurons in the MDm and in the MD–PVT transition zone. This labeling was not observed in the PVT core. The pattern of axonal labeling in the brainstem of CaMKII-injected animals differed from that of the Rbp4-Cre animals indicating that these two approaches label different subsets of L5 neurons.

To analyze the variability of axon density across the MDm, MDm–PVT transition zone, and PVT core among different animals of the same strain, we applied the CR-based parcellation approach (Fig. 7*G*) and examined mPFC projections from distinct cortical layers using Ntsr1-Cre (*n* = 4), Rbp4-Cre (*n* = 4), FoxP2-Cre (*n* = 3), vGLUT1 (*n* = 3), and CaMKIIα-driven (*n* = 5) viral labeling. Ntsr1+ (L6) projections predominantly targeted the MDm and MD–PVT transition zone, while the PVT core was nearly devoid of labeled fibers (Fig. 11*G*; one-way ANOVA, *F*_(2,9)_ = 7.555, *p* = 0.0199; Tukey's post hoc: MDm vs PVT core, *p* = 0.011, MDm–PVT transition zone vs PVT core, n.s.). In contrast, Rbp4+ (L5) projections were most concentrated in the PVT core, with a gradual increase in axon density from the MDm to the transition zone (Fig. 11*F*; one-way ANOVA, *F*_(2,9)_ = 10.82, *p* = 0.004; Tukey's post hoc: MDm vs PVT core, *p* = 0.0033, MDm–PVT transition zone vs PVT core, *p* = 0.043, MDm vs MDm–PVT transition zone, n.s.). FoxP2+ (deep L5/L6) projections exhibited widespread innervation, with higher axon density in the MDm and MD–PVT transition zone compared with the PVT core (Fig. 11*G*; one-way ANOVA, *F*_(2,6)_ = 15.0, *p* = 0.0046; Tukey's post hoc: MDm vs MDm–PVT transition zone, *p* = 0.0451, MDm–PVT transition zone vs PVT core, *p* = 0.0038, MDm vs PVT core, n.s.). vGluT1-labeled axons, representing general cortical input, showed significantly higher density in the MD–PVT transition zone compared with both the MDm and PVT core, which did not differ from each other (Fig. 11*G*; one-way ANOVA, *F*_(2,6)_ = 9.212, *p* = 0.0148; Tukey's post hoc: MDm vs MDm–PVT transition zone, *p* = 0.0241; MDm–PVT transition zone vs PVT core, *p* = 0.0221; MDm vs PVT core, n.s.). CaMKIIα-labeled projections primarily targeted the MDm and MD–PVT transition zone, with minimal PVT core innervation (Fig. 11*G*; one-way ANOVA, *F*_(2,12)_ = 25.44, *p* < 0.0001; Tukey's post hoc: MDm vs PVT core, *p* < 0.0001, MDm–PVT transition zone vs PVT core, *p* = 0.0003, MDm vs MDm–PVT transition zone, n.s.).

Together, these data demonstrate that distinct cortical layers (L5 vs L6) target different subregions of the PVT. Specifically, L5 mPFC inputs, selectively labeled by Rbp4, preferentially target the core region of the PVT. In contrast, L6 mPFC inputs, selectively labeled by Ntsr1 and extensively by FoxP2, primarily innervate the transition zone and MDm. Consistently, vGluT1-Cre tracing that labels both L5 and L6 showed highest axonal density in the transition zone, with lower but comparable labeling in the MDm and PVT core. Intriguingly, unlike vGluT1 in our experiments CAMKII promoter preferentially labeled the L6 cells as evidenced by selective innervation of the transient zone. We observed similar patterns of mPFC innervation along the anterior–posterior axis of the PVT (Fig. S8).

Since this CaMKIIα promoter construct is frequently used in other studies and is thought to label all cortical cells, we performed injections in two addition mouse strains to characterize the selectivity of CaMKIIα axonal labeling in the PVT. First, in CR-Cre mice, we injected AAV5-DIO-mCherry into the PVT to selectively label CR+ core neurons with their full dendritic arbor (Fig. 12*A–C*; see also Fig. 7). The dendritic arbor of PVT/CR+ cells were confined to the PVT core. In the same animals, we also injected the mPFC with the CaMKIIα construct to examine cortical projections (Fig. 12*C1*). All injections (*n* = 3) successfully targeted the PVT, with one mPFC injection also reaching the MO. Consistent with the data presented above (Fig. 11*D1–3*), mPFC CaMKIIα+ fibers almost completely avoided the PVT core (Fig. 12*C1–4*). Axon density showed a negative correlation with both CR and RFP intensities in the PVT (Fig. 12*B5*,*C5*), suggesting that CaMKIIα+ fibers from the mPFC are sparse in the PVT core. This does not exclude the possibility of functionally significant synapses, as reported by previous studies (Do-Monte et al., 2015; Otis et al., 2019; Aquino-Miranda et al., 2024).

**Figure 13.**
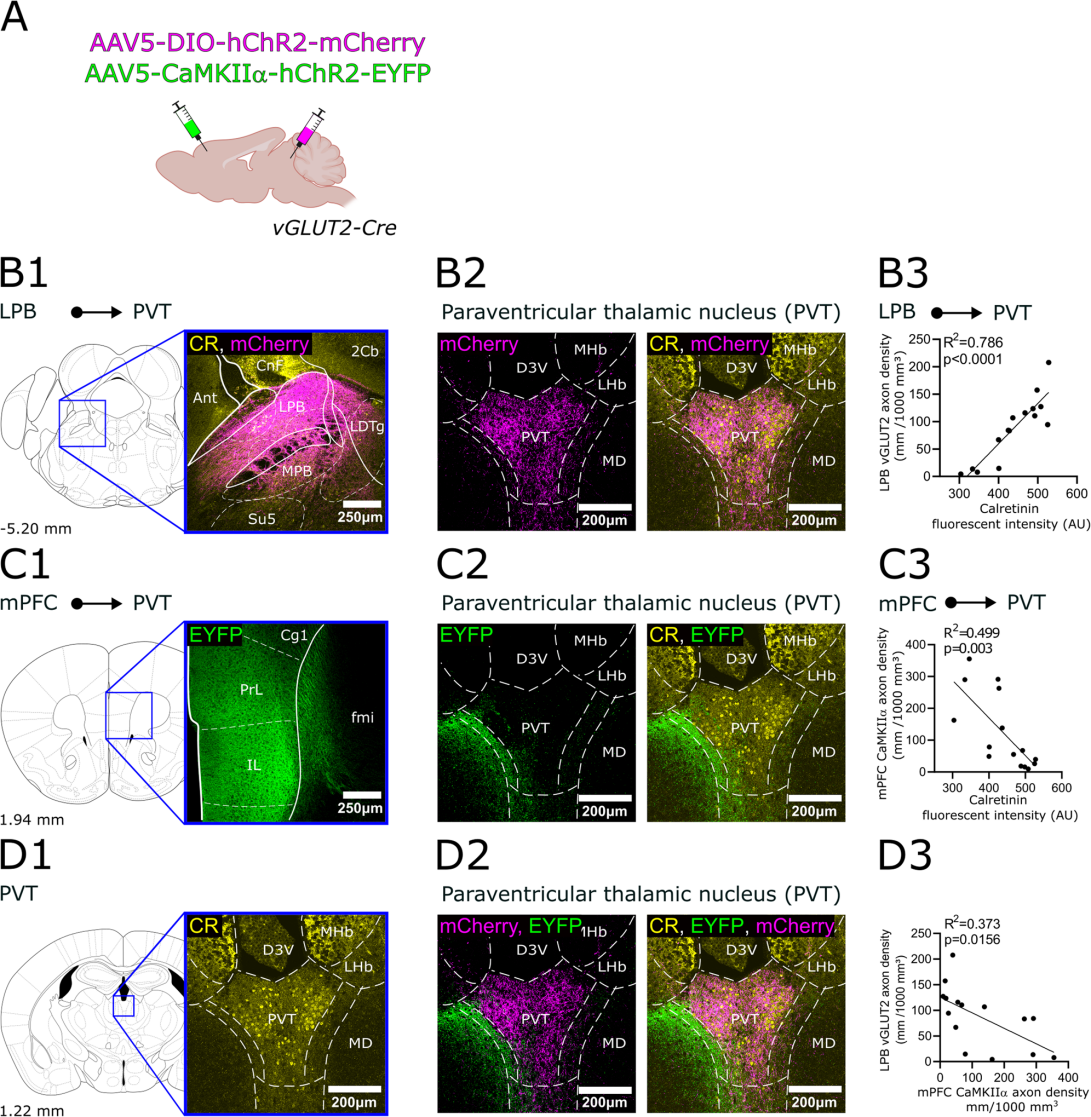
Segregation of subcortical inputs and cortical inputs labeled by CaMKIIα. ***A***, Schematic representation of the experimental setup, where Cre-dependent and CaMKIIα promoter viral constructs were injected into the LPB and mPFC, respectively, in a vGLUT2-Cre mouse. ***B1***, ***C1***, The positions of the injection sites (LPB and mPFC) in the stereotaxic atlas, along with representative images of viral expression. ***B2***, ***C2***, Distribution of axons originating from the LPB (vGLUT2) and mPFC (CaMKIIα) in the PVT/MD together with CR immunostaining. ***B3***, ***C3***, Correlations between the density of vGLUT2+ LPB and CaMKIIα+ mPFC axons and the fluorescence intensity of CR immunostaining, AU, arbitrary units. ***D1***, The position of the PVT in the stereotaxic atlas. ***D2***, Composite image showing the distribution of axons originating from the LPB and mPFC. ***D3***, Correlation between LPB axon density and mPFC axon density. 2Cb, 2nd cerebellar lobe; AAV5, adeno-associated virus serotype 5; Ant, anterior cerebellar lobe; CaMKIIα, calcium/calmodulin-dependent kinase IIα; Cg1, cingulate cortex; CnF, cuneiform nucleus; CR, calretinin; D3V, dorsal part of the 3rd ventricle; DIO, double-floxed inverted open reading frame; fmi, forceps minor; EYFP, enhanced yellow fluorescent protein; hChR2, human channelrhodopsin 2; IL, infralimbic cortex; MD, mediodorsal thalamic nucleus; MHb, medial habenula; MPB, medial parabrachial nucleus; LDTg, laterodorsal tegmental nucleus; LHb, lateral habenula; LPB, lateral parabrachial nucleus; PrL, prelimbic cortex; Su5, supratrigeminal nucleus; vGLUT2, vesicular glutamate transporter type 2.

Next, in the second experiment, we examined the convergence between the mPFC inputs labeled by the CaMKIIα virus construct and the subcortical glutamatergic afferents. We injected the CaMKIIα promoter construct into the mPFC of vGLUT2-Cre animals while AAV5-DIO-hChR2(H134R)-mCherry was injected into the LPB (Fig. 13*A*,*B1*–*D1*). All four injections (*n* = 4) successfully targeted the LPB, while one of the mPFC injections hit the MO and VO regions. Confirming our previous experiments (Fig. 8*C1–3*), axons from the LPB primarily innervated the core of the PVT (Fig. 13*B2*,*3*) while mPFC-CaMKIIα labeled axons targeted the transition zone and the MDm in a complementary fashion (Fig. 13*C2*,*3*). MO and VO injections showed a similar pattern (*n* = 1). LPB vGLUT2+ axon density and mPFC CaMKIIα+ axon density showed a significant negative correlation with each other (Fig. 13*D1–3*). Confirming previous correlation data (Fig. 8), the LPB vGLUT2+ axon density showed a positive correlation with CR fluorescence intensity (Fig. 13*B3*), while mPFC CaMKIIα+ axon density negatively correlated with CR fluorescence in the PVT (Fig. 13*C3*).

These data collectively confirm that the mPFC CaMKIIα construct does not label all cortical input to the PVT, as CaMKIIα+ fibers largely avoid the PVT core, which is innervated by mPFC Rbp4 fibers.

### Origin of cortical inputs to PVT

**Figure 14.**
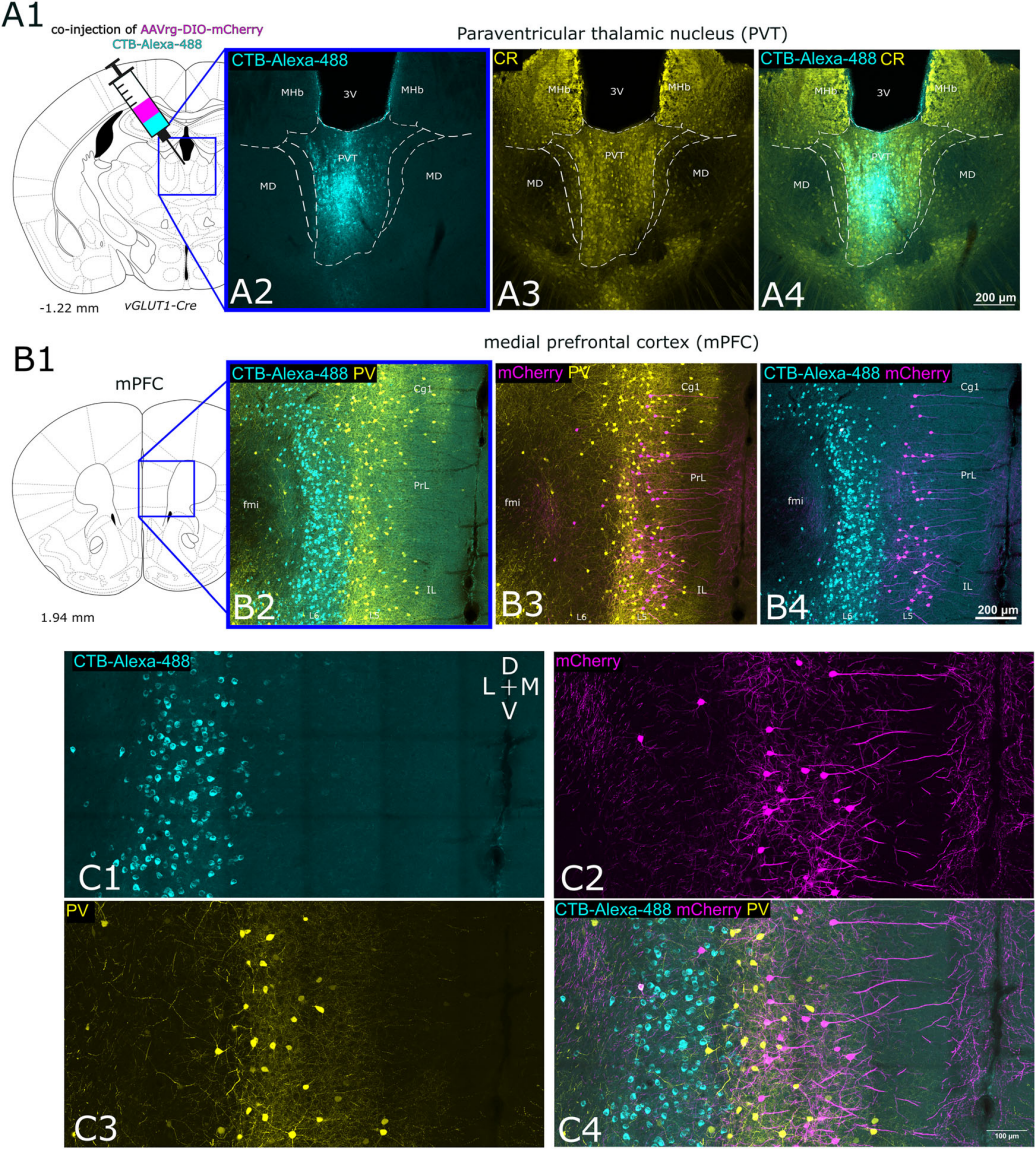
Both layer 5 and layer 6 mPFC neurons project to the PVT: distinct populations revealed by combined retro-AAV and CTB tracing. ***A1***, Schematic representation of the experimental setup, showing the coinjection of AAVrg-DIO-mCherry and CTB-Alexa Fluor 488 into the PVT of a vGluT1-Cre mouse. ***A2*–*4***, Position of the injection site in the stereotaxic atlas and representative images showing that CTB labeling (cyan) was restricted to the PVT core, as confirmed by CR immunostaining (yellow). ***B1*–*4***, Distribution of retrogradely labeled neurons in the mPFC. CTB-labeled neurons (cyan) were concentrated in layer 6, while AAVrg-DIO–labeled mCherry+ neurons (magenta) were primarily confined to layer 5. PV immunostaining (yellow) was used to delineate laminar boundaries. ***C1*–*4***, Higher magnification images showing the segregation of CTB+ and mCherry+ neurons across cortical layers. Merged panels illustrate minimal overlap between the two populations. These findings demonstrate that both L5 and L6 mPFC neurons project to the PVT and that CTB and retro-AAV label distinct corticothalamic populations. CTB, cholera toxin B subunit; CR, calretinin; D3V, dorsal part of the 3rd ventricle; DIO, double-floxed inverted open reading frame; L, lateral; M, medial; D, dorsal; V, ventral; PV, parvalbumin.

Previous anatomical studies reported that mPFC projections to the PVT arise predominantly from layer 6 neurons based on retrograde tracing with classical tracers such as cholera toxin B subunit (CTB; Hurley et al., 1991; Li and Kirouac, 2012; Aquino-Miranda et al., 2024; Ma et al., 2024). In contrast, our current data revealed a prominent layer 5 glutamatergic input to the PVT core (Fig. 11). To resolve this controversy, we carried out a direct head-to-head comparison of the two tracers in vGLUT1-Cre mice. In one cohort of animals (*n* = 5), we injected a Cre-dependent retrograde AAV (AAVrg-DIO-EYFP) into the PVT, while in a second cohort (*n* = 3) we coinjected a Cre-dependent retrograde AAV (AAVrg-DIO-mCherry) together with Alexa Fluor 488-conjugated CTB at the same PVT coordinates (Fig. 14*A*). Calretinin (CR) immunostaining confirmed that CTB spread was confined to the PVT (Fig. 14*A1–4*). As above we used parvalbumin (PV) immunostaining (Fig. 14*B1–3*)—which labels the perisomatic region of layer 5 pyramidal neurons in PrL (Mátyás et al., 2014)—to delineate the laminar boundaries (Biro et al., 2023; Nagy-Pál et al., 2023). We found that CTB-labeled neurons were concentrated in layer 6 (Fig. 14*B2*), while retro-AAV-labeled neurons were confined mainly to layer 5 (Fig. 14*B3*), with minimal overlap between the two populations (Fig. 14*B4*,*C1–3*). These findings demonstrate that CTB and retro-AAV preferentially label distinct corticothalamic pathways and confirm that both layer 5 and layer 6 mPFC neurons contribute to the innervation of the PVT. This dual-labeling approach reconciles previous discrepancies and highlights a previously underappreciated layer 5 glutamatergic projection to the PVT core.

## Discussion

In the present study, we identified the origins of subcortical excitatory and inhibitory inputs to the PVT and the layer specificity of its cortical inputs. We also mapped the regional selectivity of afferents relative to the CR+ cells of PVT. We demonstrated that the PVT receives both glutamatergic and GABAergic inputs from multiple subcortical regions, with most afferents selectively innervating the CR+ core. Additionally, we observed a layer-specific segregation of cortical inputs: L5 projections from the mPFC preferentially innervated the CR+ core, whereas L6 inputs primarily targeted the transitional zone between the PVT and the MD. These findings suggest that the CR+ cells in the core region of the PVT integrate a complex array of subcortical (excitatory and inhibitory) signals with cortical top-down L5 activity. Scarcity of L6 inputs to the PVT core is a unique feature within the thalamus. This distinctive pattern of afferentation allows the PVT CR+ core to exert unique control over multiple forebrain centers involved in affective behaviors (Hsu and Price, 2009; Penzo and Gao, 2021) via its wide-ranging collaterals (Hsu et al., 2014; Mátyás et al., 2018; Jász et al., 2025).

### Technical considerations

Our data challenge previous tract tracing data on the cortical inputs of PVT that was based on the use of CTB and CaMKIIα promoter (Hurley et al., 1991; Li and Kirouac, 2012; Otis et al., 2019; Aquino-Miranda et al., 2024; Ma et al., 2024). These studies reported a predominance of L6 input to PTV. In contrast, our data demonstrate preferential innervation of the CR+ core of PVT by L5 and dense innervation of the transitional zone by L6 neurons. This finding is supported by anterograde viral tracing in six different mouse strains and dual retrograde tracing using CTB and retrograde AAV. Collectively, these data demonstrate that both CTB and the CaMKIIα promoter preferentially label the L6 cortico-PVT pathway, leading earlier work to overestimate its importance.

### Subcortical afferents

The input regions of the PVT identified in this study align well with previous tract tracing studies (Van der Werf et al., 2002; Li and Kirouac, 2012; Vertes et al., 2022; Kirouac, 2025). However, these earlier studies did not allow the identification of the excitatory or inhibitory nature of the afferents to the PVT. Our retrograde viral tracing demonstrated that different forebrain regions provided the main inputs with exclusively a GABAergic component (BNST, ZI, TRN, LH, and DM) to the PVT whereas pure glutamatergic inputs concentrated in the caudal brainstem (LPB) and caudal hypothalamus (PSTh, SUM). These findings are supported by the limited available functional data, which indicate an excitatory influence from the LPB (Zhu et al., 2022) and inhibitory inputs from the LH (Otis et al., 2019) and ZI (Zhang and van den Pol, 2017) on PVT activity. Interestingly, the PAG, MPOA, and DpMe provided both excitatory and inhibitory inputs to the PVT, which can allow a complex, dynamic, push–pull regulation of its activity. We confirmed several input regions identified by retrograde tracing using anterograde viral tracing. In addition to previously described subcortical inputs, our study found the MPL as a novel source of PVT innervation, expanding the known network of structures that communicate with this thalamic region.

### Nuclear boundaries of the PVT

Soma-dendritic organization of CR+ neurons in PVT clearly delineated a central core region of PVT. Most cortical and subcortical inputs respected this boundary confirming its functional relevance. Utilization of CR as a neurochemical marker is supported by previous studies demonstrating that CR expression defines functionally distinct thalamic subpopulations (Arai et al., 1994; Mátyás et al., 2018; Gao et al., 2023; Jász et al., 2025). Mapping of CR+ cells and their inputs in the human thalamus (Mátyás et al., 2018) demonstrated a similar regional arrangement to that observed mice, indicating that the organization of the PVT is evolutionarily conserved. Inhibitory inputs from TRN and BNST afferents innervated both the PVT core and extended into the medial MD (Fig. 9). For the TRN, our injections could not be restricted to the TRN sector targeting only the PVT, and they spread to the neighboring regions containing MD-projecting TRN cells. For the BNST, it remains to be established whether distinct GABAergic cell populations in the posteromedial BNST target the PVT and MD or whether this GABAergic input can coordinate the activity of MD and PVT.

### Layer-specific segregation of cortical afferents in PVT

We found the segregation of L5 and L6 afferents between the CR+ PVT core and the transition zone. These observations are corroborated by the distinct cortical cell populations labeled in our Cre-lines (Rbp4 and Ntsr-1; Fig. 11; DeNardo et al., 2015; Tasic et al., 2016; Hoerder-Suabedissen et al., 2018; Babiczky and Matyas, 2022; Hádinger et al., 2023), the morphological characteristics of the cortical terminals and retrograde tracings. Specifically, for the Rbp4+ L5 terminals in the PVT core, we found globular architecture and while Ntsr-1 L6 terminals in the transition zone were of drumstick-like morphology (Fig. S6; Bourassa and Deschênes, 1995; Deschênes et al., 1998; Hoerder-Suabedissen et al., 2018). The neuronal markers FoxP2 and vGluT1, which label both L5 and L6 in the mPFC, provided input to both the core and the transition zone, as expected, whereas CaMKIIα promoter visualized predominantly the L6 fibers to the transient zone. Dual retrograde tracing (CTB+ retro-AAV) in vGluT1-Cre mice revealed that CTB preferentially labels L6 neurons, whereas retro-AAV visualized mainly L5 neurons—explaining the L6 dominance in earlier reports and uncovering a previously underappreciated L5 glutamatergic pathway to the PVT core. Together, our results demonstrate that both cortical layers innervate the PVT with clear subregion specificity.

L6 input is regarded as a canonical input to all thalamic nuclei (Acsády, 2022) whereas L5 innervates only a selected set of so-called “higher order” nuclei (Sherman and Guillery, 2013; Sherman, 2016). L5 input, in general, is regarded as a “driver” input that strongly affects firing activity of its target, whereas the L6 corticothalamic pathway is considered modulatory (Sherman, 2016). These anatomical data indicate a strong L5 excitatory influence of the mPFC over PVT activity. Indeed, optogenetic silencing of mPFC-PVT projections has been shown to significantly reduce PVT neuronal firing in vivo (Do-Monte et al., 2015), and complementary work has demonstrated that in vivo stimulation of PL→PVT projections elicits robust excitation of PVT neurons (Otis et al., 2019). However, recent results indicate a predominantly inhibitory influence of the PrL on the PVT via feedforward inhibition mediated by the TRN (Aquino-Miranda et al., 2024; Ma et al., 2024). We propose that direct L5 excitation and indirect inhibition via the TRN are not mutually exclusive since both the L5 and the L6 of the mPFC have been reported to innervate the TRN sectors that project to the PVT (Hádinger et al., 2023; Aquino-Miranda et al., 2024; Ma et al., 2024). However, which of these effects is stronger in any given experiment depends on the exact type of cortical labeling. When we performed anterograde tracing using the CaMKIIα promoter [AAV5-CaMKII-ChR2(H134R)-EYFP] vector utilized in two studies describing mainly feedforward inhibition between mPFC and PVT (Aquino-Miranda et al., 2024; Ma et al., 2024), we found that the labeled fibers preferentially targeted the transition zone and almost completely avoided the CR+ core (Figs. 11–13). Our data thus demonstrate that this specific viral construct label PVT-projecting L5 subpopulations only with low probability despite the fact that CaMKIIα mRNA is ubiquitously expressed in cortical excitatory neurons. Consequently, direct L5 mPFC-PVT excitation will not be observed using this viral vector, and PVT cells will receive predominantly feedforward inhibition via the TRN upon mPFC activation, which is consistent with these studies (Aquino-Miranda et al., 2024; Ma et al., 2024). In summary, these data together demonstrate a complex top-down control of mPFC over PVT, characterized by layer- and region-specific selectivity, as well as the engagement of feedforward inhibition.

The organization pattern of PVT, as revealed here, is exceptional among thalamic nuclei. Most thalamic nuclei receive excitatory and inhibitory inputs from a relatively limited number of regions (Acsády, 2022). Cortical L5 driver inputs and vGLUT2+ subcortical inputs have not been found to converge extensively (Rovó et al., 2012; Groh et al., 2014; Casas-Torremocha et al., 2022). In contrast, we found that a large number of subcortical excitatory and inhibitory inputs converge on a specific sector of the medial thalamus, the CR+ core region of the PVT, where they intermingle with cortical L5 afferents. Notably, some afferent regions targeting the PVT core provide both excitatory and inhibitory inputs, a phenomenon that, to our knowledge, has been directly demonstrated only in one case in the thalamus so far (Bősz et al., 2025). The integration of diverse subcortical and cortical signals enables the PVT to play a pivotal role in stress adaptation, homeostatic regulation, and motivational behaviors (McGinty and Otis, 2020; Kirouac, 2025). Future research should explore how these distinct inputs contribute to PVT-dependent cognitive and affective processes, with a particular emphasis on functional connectivity across stress, arousal, and reward circuits.
